# Scale‐dependent effects of host patch traits on species composition in a stickleback parasite metacommunity

**DOI:** 10.1002/ecy.3181

**Published:** 2020-10-01

**Authors:** Daniel I. Bolnick, Emlyn J. Resetarits, Kimberly Ballare, Yoel E. Stuart, William E. Stutz

**Affiliations:** ^1^ Department of Integrative Biology University of Texas at Austin Austin Texas 78712 USA; ^2^ Office of Institutional Research Western Michigan University Kalamazoo Michigan 49008‐5253 USA; ^3^ Present address: Ecology and Evolutionary Biology & Institute of System Genomics University of Connecticut Storrs Connecticut 06269 USA; ^4^ Present address: Center for the Ecology of Infectious Disease Odum School of Ecology University of Georgia Athens Georgia USA; ^5^ Present address: Ecology and Evolutionary Biology University of California Santa Cruz Santa Cruz California 95064 USA; ^6^ Present address: Department of Biology Loyola University Chicago Illinois 60660 USA

**Keywords:** diet, helminth, infection, infracommunity, macroparasite, metacommunity, spatial scale, threespine stickleback

## Abstract

A core goal of ecology is to understand the abiotic and biotic variables that regulate species distributions and community composition. A major obstacle is that the rules governing species distributions can change with spatial scale. Here, we illustrate this point using data from a spatially nested metacommunity of parasites infecting a metapopulation of threespine stickleback fish from 34 lakes on Vancouver Island, British Columbia. Like most parasite metacommunities, the composition of stickleback parasites differs among host individuals within each host population, and differs between host populations. The distribution of each parasite taxon depends, to varying degrees, on individual host traits (e.g., mass, diet) and on host‐population characteristics (e.g., lake size, mean host mass, mean diet). However, in most cases in this data set, a given parasite was regulated by different factors at the host‐individual and host‐population scales, leading to scale‐dependent patterns of parasite‐species co‐occurrence.

## Introduction

A classic dichotomy in ecology is whether communities are deterministic co‐occurring sets of species (Clements 1916) or collections of many species following an independent set of stochastic rules (Gleason 1926). Metacommunity theory (Leibold et al. 2004, Leibold and Chase 2017) bridges the gap between these competing visions by considering relative roles of determinism and stochasticity at various spatial scales on a fragmented landscape. When species have similar filters governing dispersal to new patches and persistence within patches, they will tend to co‐occur and form a more deterministic community (the Clementsian model). A particular form of this deterministic community assembly arises from between‐species interactions, for instance, if competing species exclude each other or symbionts require each other’s presence (the Eltonian view of ecology). If, instead, each species’ distribution is stochastic, or is subject to independent filters, then communities will be composed of independently distributed species (the Gleasonian model). Thus, a key question in metacommunity theory is, what abiotic and biotic filters regulate species’ dispersal or within‐patch dynamics? Then, do these filters affect multiple species in a related manner, thus creating deterministic rather than stochastic species assemblages? Lastly, do the answers to these questions depend on the spatial scale at which one defines a community? Here, we address these questions using a multispecies metacommunity of parasites.

Parasite communities are an ideal system to apply metacommunity theory (Lima et al. 2012, Mihaljevic 2012, Seabloom et al. 2015, Borer et al. 2016). The ideas underlying the metacommunity concept have long been embraced by parasitologists, though with different terminology (Appleton 1983, Esch 1994, Kuris and Lafferty 1994, Lafferty et al. 1994, Bush et al. 1997). Metacommunity theory emphasizes the processes of dispersal between and persistence within patches (Leibold et al. 2004, Leibold and Chase 2017). These same themes are developed within parasite ecology, using the terminology (1) host‐encounter filters and (2) host‐compatibility filters (Combes 2001). From the parasite point of view, individual hosts are transient habitat patches that contain a community of parasites, an “infracommunity” per parasite ecology (Bush and Holmes 1986, Poulin 1996, Bush et al. 1997, Poulin 1997, Combes 2001). The host population thus represents a single parasite metacommunity (a “component community” in parasite ecology) that persists because parasites are transmitted from infected to uninfected individuals. This small‐scale metacommunity is often nested within a larger metacommunity which is an assemblage of many distinct host populations (Combes 2001). At either spatial scale, parasites must disperse between patches (host individuals or populations) and persist within those patches long enough to produce successfully dispersing progeny (Seabloom et al. 2015). These processes of dispersal and persistence should depend on host (patch) characteristics. Accordingly, there are many published studies of parasite metacommunities and how their composition depends on abiotic and biotic variables (Ebert et al. 2001, Mihaljevic 2012, Raeymaekers et al. 2013, Richgels et al. 2013, Dallas and Presley 2014, Seabloom et al. 2015, Borer et al. 2016, Cirtwill et al. 2016, Johnson et al. 2016, Hayward et al. 2017).

**Fig. 1:**
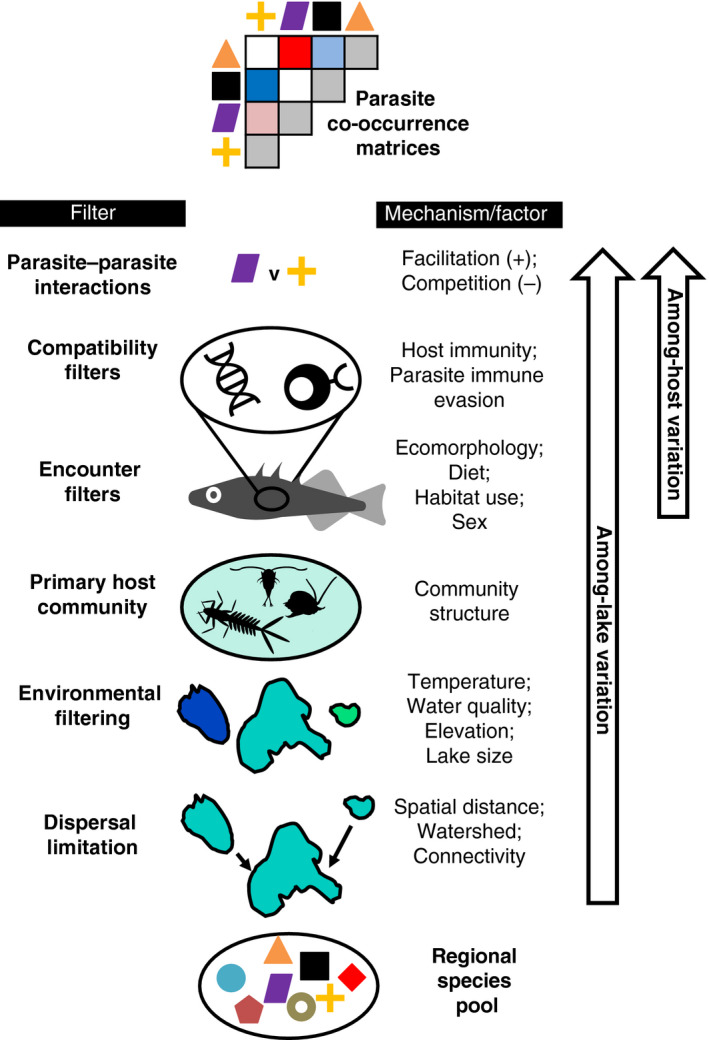
A schematic of factors contributing to parasite species’ distribution, co‐occurrence, and metacommunity composition. We list examples of host‐individual and host‐population traits (right column) underlying general filters (left column). Arrows indicate the spatial scale at which the factors listed in the diagram are likely to act. [Color figure can be viewed at wileyonlinelibrary.com]

The processes that generate dispersal and persistence filters are expected to differ by spatial scale in a parasite metacommunity (Fig. 1; Combes 2001). For reasons elaborated below, some filters will act among both host individuals and host populations, and others might be relevant only at one scale. Such scale‐dependent assembly rules might cause a parasite metacommunity to be more deterministic at one spatial scale and more stochastic at another. Or, scale‐dependent rules will cause different combinations of deterministic species co‐occurrence across scales. For example, there has been a long history of investigating the assembly rules of spatially nested metacommunities of parasites inhabiting snail hosts (Appleton 1983, Esch and Fernandez 1994, Kuris and Lafferty 1994, Lafferty et al. 1994, Bush et al. 1997). These studies have found that infracommunities (i.e., within host individuals) are structured by a combination of host body size and parasite competitive interactions (leading to negative parasite co‐occurrence). In contrast, the component communities (i.e., within host populations) are structured by environmental variables and heterogeneity (leading to positive parasite co‐occurrence; see Esch and Fernandez 1994 for review). Our study builds on such past work by investigating how numerous host and environmental characteristics (host sex, size, diet, morphology, and genotype; habitat elevation, isolation, and size) contribute to patterns of co‐occurrence across parasite species, and evaluating which effects are scale independent or scale dependent.

### Scale‐independent factors

Some dispersal and persistence filters should act similarly across scales. Consider parasites with complex life cycles, where a focal host species must ingest an infected prey (intermediate host) to be exposed to a parasite. Assuming the life cycle of the parasite is relatively fixed across a host metapopulation, host diet should have similar effects on infection risk at both spatial scales (among individuals, and among populations). Host populations often show ecomorphological and behavioral variation between individuals, leading to within‐population variation in diet (Bolnick et al. 2003). This diet variation can cause host individuals to encounter different prey‐acquired parasites at different rates (Hausfater and Meade 1982, Lafferty 1992, Wilson et al. 1996, Hutchings et al. 2003, Skartstein et al. 2005, Johnson et al. 2009, MacColl 2009, Johnson and Thieltges 2010, Stutz et al. 2014). For example, limnetic‐feeding stickleback eating planktonic copepods are more likely to ingest copepod‐transmitted *Schistocephalus solidus* cestodes, compared to benthivorous individuals in the same population (Stutz et al. 2014). At the host‐population scale, mean diet should predict exposure to and infection by diet‐transmitted parasites. Thus, we expect ecologically driven dispersal filters to have similar effects across scales. Likewise, host body size is widely known to be positively related to infection load and diversity, because larger hosts may be more active foragers (higher exposure rate) or older (greater cumulative exposure risk). Either mechanism might generate positive size–infection correlations both among individuals and among populations.

### Among‐host‐population factors

A unique and fascinating feature of parasite metacommunities is that the host “patch” actively changes in order to kill or expel colonizing parasites. The capacity to resist parasites can be acquired by individuals after initial exposures, via induced immune responses, most notably vertebrates’ adaptive immune responses that protect against subsequent reinfection. Populations also evolve resistance to their most severe or commonly encountered parasites, generating among‐host‐population variation in resistance, which in turn reduces the prevalence of formerly successful parasites (Maizels and Yazdanbakhsh 2003, Viney et al. 2005, Schmid‐Hempel 2009). Because populations evolve, but individuals do not, evolution generates a uniquely population‐scale source of variation in encounter filters (evolved avoidance behaviors) or compatibility filters (evolved host immunity) that apply to most or all hosts in a population (Best et al. 2009, Berenos et al. 2010, Gilman et al. 2012, Luijckx et al. 2013). Thus, host or parasite local adaptation will tend to generate heritable differences in parasite communities (Hoeksema and Forde 2008) but only at the among‐population spatial scale.

This evolved immunity can undermine the relationship between encounter filters and infection rates. To illustrate this point, consider the case of *Schistocephalus solidus* cestodes, which infect stickleback when they consume cyclopoid copepods. All else being equal, we expect more limnetic‐feeding stickleback that consume more copepods to be more infected (as noted above). But, at the population scale limnetic stickleback may then evolve greater resistance to *S. solidus*, resulting in low rates of successful infection despite a high ecological risk of exposure (Stutz et al. 2014). As a result, populations that were historically most at risk of a given parasite infection might today be most resistant and hence least infected.

Herd immunity generates another uniquely population‐scale effect. When a large enough fraction of hosts in a population is immune to a given parasite, even susceptible individuals are protected because their exposure rate declines. Thus, the effect of among‐individual diet variation on infection risk might be obscured when enough individuals are resistant.

Different host populations may inhabit substantially divergent abiotic and biotic settings, giving rise to large differences in parasite colonization and persistence. Abiotic conditions (temperature, elevation, salinity) differ across a landscape and play a major role in structuring among‐host‐population parasite communities (Ebert et al. 2001, Richgels et al. 2013, Dallas and Presley 2014, Cirtwill et al. 2016, Moss et al. 2020). For example, the cestode *S. solidus* fails to hatch in brackish water, and so is unique to freshwater rather than marine stickleback. Likewise, ecological communities can differ across a landscape, changing parasite infection rates via the abundance of suitable and unsuitable intermediate, alternative, and terminal hosts. One example is the widely studied dilution effect, in which ineffective alternative hosts soak up parasite propagules without subsequent reproduction and retransmission (Johnson and Thieltges 2010, Becker et al. 2014). In the case of stickleback, the difference between high and low prevalence of the *S. solidus* cestode (that uses piscivorous birds as a terminal host) can be caused by a single breeding pair of loons (Heins et al. 2011). More generally, spatial variation in the diversity and identity of terminal hosts can generate among‐population differences in multiple parasite species that use those terminal hosts (Hechinger and Lafferty 2005). A caveat here is that although abiotic and biotic differences between host populations are obvious and large, there may be appreciable abiotic and biotic variation within a supposedly well‐mixed population (Maciejewski et al. 2020), which may yield comparable effects at the within‐population scale.

### Within‐host‐population factors

Filters might also generate metacommunity structure only among individual hosts within a population. Sexual dimorphism in infection is common in natural populations (Reimchen and Nosil 2001). Males and females systematically differ in diet (Shine 1989) and immunity (Rolff et al. 2009), which should contribute to within‐population variation in infection. Assuming most host populations have an equal sex ratio, parasite metacommunity structure due to sexual dimorphism should be restricted to within‐population scales. We therefore expect that the magnitude and direction of sexual dimorphism may influence the direction and magnitude of between‐sex differences in infection rates within populations, but not shift overall parasite communities among populations.

Parasite–parasite interactions are another strictly within‐host phenomenon. Co‐occurring parasites within an individual can inhibit each other’s viability via competition for shared resources, or by activating host cross‐immunity (Holmes 1990, Sousa 1990, Esch and Fernandez 1994, Kuris and Lafferty 1994, Lafferty et al. 1994, Jolles et al. 2008). Conversely, parasites are often immunosuppressive for their own benefit, generating a public good (for other parasites) that facilitates coinfection (Telfer et al. 2010). These competitive or facilitative interactions necessarily act within individual hosts.

To summarize, disease ecologists have long recognized that abiotic variables, community features, and host traits should structure parasite metacommunities. Some of these factors should act primarily at the among‐host‐individual scale (e.g., sexual dimorphism) and others at the among‐population scale (e.g., evolved or herd immunity, abiotic conditions, dilution effects). Still other factors should act similarly across spatial scales (e.g., diet increasing infection risk from prey‐transmitted parasites). These scale‐dependent and independent effects should then dictate which parasite species tend to co‐occur, or not, and to what extent the parasite metacommunity is structured by deterministically co‐occurring (or, mutually exclusive) species, or independently distributed taxa.

To test the expectations outlined above, we document the metacommunity structure of a multispecies parasite assemblage infecting threespine stickleback (*Gasterosteus aculeatus*). This is an appealing study system because stickleback populations (e.g., in separate lakes) differ nonrandomly in parasite community diversity (Eizaguirre et al. 2011), composition (Poulin et al. 2011, Stutz et al. 2015), and infection intensity (Pennycuick 1971, Weber et al. 2017). Differences in infection among stickleback populations have been shown to be temporally stable (Weber et al. 2017, Young and MacColl 2017), and have been linked to differences between stickleback populations in immune genotype (Matthews et al. 2010, Eizaguirre et al. 2012*b*, Stutz and Bolnick 2017), diet (Matthews et al. 2010), and abiotic conditions (e.g., salinity; Simmonds and Barber 2016). Within populations, individual stickleback infection is correlated with individual diet, ecomorphology, sex, and immune genotype (Reimchen 1997, Reimchen and Nosil 2001, Wegner et al. 2003, Matthews et al. 2010, Eizaguirre et al. 2012*a*, Stutz et al. 2014, Stutz and Bolnick 2017). However, these prior studies of stickleback infections have tended to focus on one or a few variables at a time, and are generally restricted to a single spatial scale. Perhaps because of this scale‐limited scope, there have been conflicting conclusions among the studies cited above.

Here, we identify abiotic, genetic, phenotypic, and ecological features of host individuals and host populations that help explain among‐individual and among‐population variation in parasite composition. We identify scale‐independent and scale‐dependent factors influencing parasite community composition. A related paper (Bolnick et al. 2020) uses these same data to examine scale dependence of factors regulating parasite species richness, an emergent property of the processes considered here.

## Methods

### Collection

In June 2009, we collected stickleback from 34 lakes in nine watersheds on Vancouver Island, British Columbia, Canada (details in Appendix S1: Table S1, Fig. S1), in the historical lands of the Kwakwaka'wakw First Nations. Collection and animal handling were approved by the University of Texas IACUC (07‐032201) and a Scientific Fish Collection Permit from the Ministry of the Environment of British Columbia (NA07‐32612). We also sampled from eight streams and five anadromous populations in estuaries, but for most analyses here we focus on lake population data. Sites were chosen nonrandomly to sample a broad array of lake types within a small geographic region. We placed unbaited 0.5‐cm gauge wire minnow traps along ~200 m of shoreline in 0.5–3‐m deep water. We obtained 60–100 fish per site (Appendix S1: Table S1). Fish were immediately euthanized in MS‐222 and preserved in 10% buffered formalin after cutting a fin clip into ethanol for DNA. Specimens were rinsed and stored in 70% isopropyl alcohol after staining with Alizarin Red.

### Data acquisition 1: parasite infections

We counted macroparasites (helminths, crustaceans, molluscs, and microsporidia) in each fish with a stereodissection microscope. We scanned the skin, fins, and armor plates, and then the buccal cavity and gills. We then dissected the body cavity and organs (liver, swim bladder, gonads, eyes) and opened the digestive tract. Parasites were identified to the lowest feasible taxonomic unit (typically genus). For abundant gill parasites, we counted parasites only on the right side. For each taxon, we calculated per‐population infection prevalence (proportion of fish infected) and abundance (mean number of parasites per fish) following Bush et al. (1997), and confidence intervals of proportions following Newcombe (1998).

### Data acquisition 2: stickleback morphology

We quantified stickleback ecomorphology, which is known to covary with individual diet within populations (Robinson 2000, Snowberg et al. 2015) and among populations (Lavin and McPhail 1985, Lavin and McPhail 1986). Before necropsy, we weighed all fish to 0.01 g and used digital calipers to measure external body dimensions (in millimeters): standard length, body depth, and body width at the pectoral fins. For a subset of ~30 individuals per population, we measured trophically important traits: gape width, gill raker number, and length of the longest gill raker. We inspected gonads via dissection to determine sex. Linear measurement data were log transformed and size‐standardized by regression on log standard length. For reference, stickleback body size is expected to play a major role in generating variation in parasite diversity and composition, and varies both within and among host populations (the latter explaining 45.8% of variance in log mass in this data set).

### Data acquisition 3: stickleback diet

For a random subset of 28 populations, we analyzed stickleback stomach contents for recent diet. Previous studies have shown that individual sticklebacks’ stomach contents are indicative of long‐term diet as inferred from stable isotopes, morphology, and feeding observations in the wild (Snowberg et al. 2015). We removed stomachs from the same fish measured for morphology, and identified the presence/absence of each prey taxon to the lowest feasible taxonomic level (typically family). For analysis, we binned prey taxa into functional groups (benthic or limnetic) and calculated the proportion of benthic prey in each fish’s stomach. For each population, we calculated the average proportion of benthic prey across the sampled individuals. This metric is strongly correlated with the major axis of dietary variation identified by Non‐metric Multi‐Dimensional Scaling (NMDS) analysis (Bolnick and Ballare 2020), but we focus on the more biologically intuitive metric here.

### Data acquisition 4: stickleback genetic diversity

To quantify the effect of host genetic variation on parasite distributions, we used ddRADseq (Peterson et al. 2012) to obtain single nucleotide polymorphism (SNP) genotypes from a subsample of 12 fish from each of 31 lakes (Table S1 in Bolnick et al. 2020). Protocols, bioinformatics steps, and SNP filtering are exactly as described in Stuart et al. (2017). The result was a matrix of genotype scores for 175,350 SNPs in 336 fish (averaging 107,698 SNPs per individual; 36 individuals were dropped because of poor sequence coverage). We calculated genome‐wide heterozygosity for each fish, and between‐population genetic distances (Weir‐Cockerham unbiased *F*_ST_).

### Data analysis

The data for this study are archived online (Bolnick and Ballare 2020, and Data S1 with associated Metadata S1). We began by testing for nonrandom co‐occurrence between parasite species. We considered co‐occurrence at the level of host individuals within populations, then among host populations, then tested whether co‐occurrence is similar across these scales. Next, we tested for individual host or host‐population characteristics that might act as dispersal or persistence filters that affect parasite species distributions. Last, we tested whether different parasite species are subject to similar filters, consistent with our inferences about their co‐occurrence or lack thereof.

#### Analysis 1: Do certain pairs of parasite species tend to co‐occur within hosts?

We estimated a co‐occurrence matrix between parasite species within each of the 33 host populations. This matrix measures the tendency for pairs of parasite species to infect the same host individuals (Fenton et al. 2010). We calculated Spearman rank correlations between all pairs of common parasite taxa (i.e., infecting >5 fish within a population). The Holm‐adjusted *P*‐values from these rank correlations test the null hypothesis that the pairwise combinations of parasite species are independently distributed among individual hosts within a given host population.

Patterns of co‐occurrence between parasite taxa might be inconsistent between host populations. For each possible pairwise comparison of populations, we calculated a Mantel correlation between their respective co‐occurrence matrices. A significant positive correlation implies that similar parasite combinations co‐occur in both host populations (Poulin 2007, Presley 2011, Meynard et al. 2013), rejecting the null hypothesis that two populations have independent parasite co‐occurrence. Conversely, we also tested whether two lakes’ co‐occurrence matrices are compellingly different, using *testCov* (*HDtest* package in R). For this test, the null hypothesis is that the two lakes share the same covariance architecture, and any observed difference is simply a result of sampling error. For both the testCov and Mantel tests, we used only parasite taxa found in both populations being compared.

In many host–parasite systems, infection intensity increases with host size or age. Thus, co‐occurrence between parasite species might simply reflect shared dependence on host size. To evaluate this simple explanation, we iterated through all possible pairs of parasite taxa *i* and *j*, and used a negative binomial general mixed model to test whether the intensity of parasite *i* was predicted by population (a random intercept), log host mass, the intensity of parasite *j*, and interactions between host population and log mass or parasite *j* (random slopes). For these analyses we excluded host populations where either parasite infected fewer than five individuals, to minimize difficulties with model convergence.

#### Analysis 2: Do parasite species tend to co‐occur at the scale of host populations?

If patterns of co‐occurrence are scale dependent, parasite co‐occurrence matrices should differ within vs. between host populations. To test this, we calculated the mean parasite abundance (sensu Bush et al. 1997) for each taxon in each lake. We then calculated the Spearman correlation between the mean abundances of each pair of parasite taxa, at the host‐population level. We excluded host populations where either parasite infected fewer than five individuals. Each pairwise comparison was tested against a null hypothesis of independence.

Next, we tested whether within‐population co‐occurrence and between‐population co‐occurrence matrices are similar (against a null hypothesis that they are independent). For each host population, we used a Mantel test to compare the focal population's individual‐level co‐occurrence matrix (estimated in Analysis 1) vs. the between‐population co‐occurrence matrix (preceding paragraph). A significant positive correlation would suggest that the processes generating co‐occurrences are similar across scales. As a mirror image analysis, we used the *testCov* function to check for significant dissimilarity between each within‐lake co‐occurrence matrix compared against the among‐lake matrix (the null hypothesis being identical correlations).

#### Analysis 3: What traits of host individuals predict within‐fish parasite community structure?

We used negative‐binomial mixed model GLMMs (Generalized Linear Mixed Models) to relate each parasite taxon’s abundance as a function of host traits, with host population as a random intercept and random slope. For the full data set of all hosts, we tested for sex and mass effects. For the 30 individuals per population with detailed morphological data we tested for effects of sex, mass, gill raker number, gill raker length, and gape width. For the subset of populations with diet data, we ran similar GLMMs adding host diet NMDS axis 1 and 2 as model predictors (cumulatively explaining 26.6% of overall diet variation), again with host population providing a random intercept and slope. Sample sizes for these models are given in Appendix S1: Table S1.

Last, we tested for individual‐host genotype effects on parasite prevalence using a genome‐wide association study (GWAS). For each SNP, we used a binomial GLM to test whether the presence of infection by a given parasite depended on individuals’ genotypes at that SNP, with host population as a fixed effect to control for among‐population covariation in infection and genotype. Using false discovery rate correction, we iterated this analysis across all SNPs that were scored on at least 25% of the sampled fish and had a region‐wide minor allele frequency exceeding 5% (for populations where the focal SNP was polymorphic). We only analyzed parasites found in at least five populations.

#### Analysis 4: What features of host populations predict across‐site parasite community structure?

We used variation partitioning (Borcard and Legendre 2002, Cottenie 2005, Peres‐Neto et al. 2006, Logue et al. 2011) to find abiotic and biotic factors that contribute to among‐host‐population variation in parasite metacommunity composition and estimate how much variation in multispecies community composition could be partitioned to among‐host‐population spatial distance, genetic distance (*F*_ST_), ecomorphology, and environment (for this one analysis we included stream populations in addition to the lakes examined elsewhere in this paper). We excluded two lakes and one stream for which we did not obtain sufficient ddRADseq sequence data (Browns Bay Lake, Farewell Lake, Farewell Stream). We also excluded two sites from a separate island (Quadra—Village Bay Lake and Village Bay Stream), whose geographic distance had high leverage on the distance effect estimate, leaving a total of 36 sites. We were interested in two spatial distance matrices: a “fish swims” spatial distance calculated along tributaries that connect two sites, and over‐land Euclidean distance. For spatial and genetic data, distance matrices were converted into rectangular data through principal coordinates of neighborhood matrix (PCNM), which computes a principal coordinate analysis using a truncated distance matrix. For PCNM, we used the function pcnm() in the package vegan and extracted eigenvectors with positive Moran’s index of autocorrelation (Dray et al. 2006). We then ran redundancy analysis (RDA) on each component (spatial, genetic, ecomorphology, environment) to determine if that component significantly explained Hellinger‐transformed parasite community data. Overland Euclidean distance was not significant and not included in variation partitioning analyses. For all other components, we ran forward selection on that component to determine which variables to include. We retained log gape‐width residuals and condition for ecomorphology and retained habitat and maximum depth for environment. We then ran the function *varpart*() in the package vegan to partition variation in community data between these four components.

Next, we examined each parasite taxon separately to identify lake‐level abiotic and host phenotypic traits associated with each parasite taxon’s prevalence. Using lake as the level of replication, we used binomial GLMs to regress parasite prevalence against site characteristics (lake area and elevation) and host‐population characteristics (means of fish mass, gill raker length, and gape width). For the subset of lakes with diet data, we re‐ran these analyses, adding the top two NMDS axes of diet variation as independent variables.

To evaluate genetic contributions to among‐population variation in infection rates, for each SNP we used a binomial GLM to test whether each parasite taxon’s prevalence was a function of that SNP’s allele frequency (using population as the level of replication), with watershed as a covariate. We calculated *q*‐values to adjust for multiple comparisons.

#### Analysis 5: Do co‐occurring parasites depend on host and population traits in similar ways ?

Co‐occurrence between parasite species (documented by Analyses 1 and 2) are likely to arise from different species’ shared dependence on particular dispersal or compatibility filters (evaluated separately for each species in Analyses 3 and 4). For our final analysis, we tested whether the various parasite taxa exhibit similar responses to host and population traits. The alternative is that each species follows an independent set of rules, and depends on its own unique set of host traits. We took the effect‐size estimates (*Z* scores) from the GLMs in Analysis 4, which represent the effect of lake characteristics and host‐population trait means on each parasite taxon. We then calculated the correlation between these effect sizes for each pair of parasite taxa. These correlations generated a “co‐dependence” matrix expressing the similarity, for each pair of parasite taxa, in their dependence on host and environmental traits (at the host‐population scale). We restricted this analysis to the among‐population scale because each lake exhibited its own unique pattern of among‐individual parasite co‐occurrence and trait dependence.

## Results

**Fig. 2:**
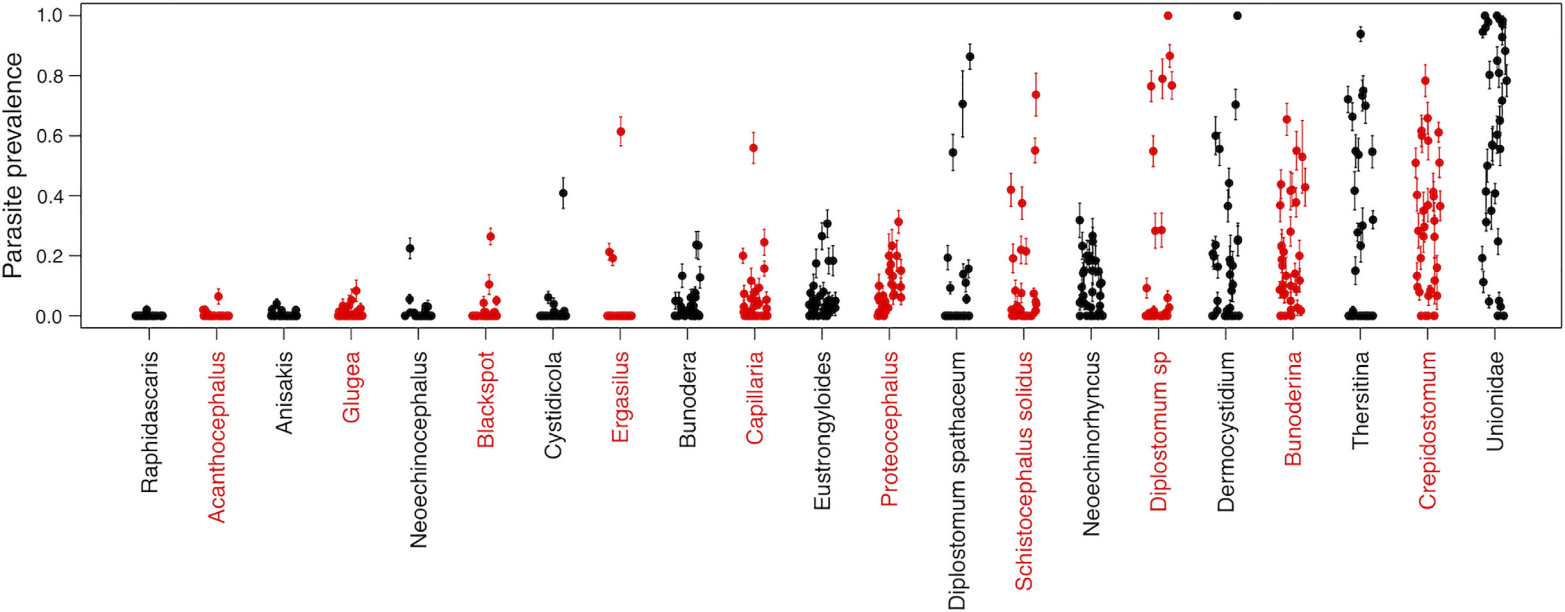
Variation in parasite prevalence among 34 lake populations of threespine stickleback. Each point represents the proportion of fish infected by the focal parasite, with standard error bars. Parasites are ordered along the x‐axis from least to most common in the metacommunity. [Color figure can be viewed at wileyonlinelibrary.com]

We observed striking variation in the prevalence and types of parasite infections among stickleback populations and among individual fish within populations (Fig. 2). For example, parasitic bivalve larvae (Unionidae) range from as low as 0% prevalence (e.g., in Higgens Lake, 95% confidence interval [CI] 0–5%), to 100% prevalence (e.g., in Little Mud Lake, 95% CI 93–100%). Within a given lake, some individuals had zero Unionidae, whereas other fish had up to 120 covering their gills. Although only a few taxa spanned such a wide range, every parasite had highly significant among‐population differences in prevalence (binomial GLMs, all *P* < 0.0001). We therefore used these data to evaluate which host and site traits predicted the distribution of individual parasites, and parasite–parasite co‐occurrence across spatial scales.

### Analysis 1: Parasites co‐occur within hosts, but these covariances differ between lakes

**Fig. 3:**
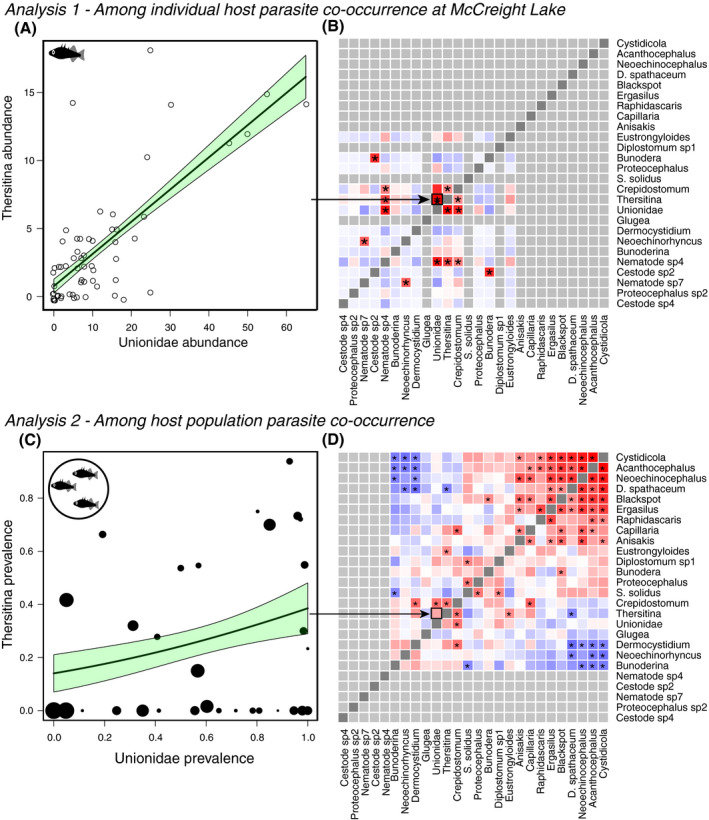
Parasite co‐occurrence among individual hosts (Analysis 1) and among host populations (Analysis 2). (A) A scatter plot of the correlation between the abundance of *Thersitina* and *Unionidae* infections within McCreight Lake (*N* = 60 fish). Points represent host individuals. There are overlapping points at (0, 0). (B) The parasite species co‐occurrence network observed among individual stickleback. Red and blue shaded cells show positive and negative covariance, respectively; asterisks denote significant Spearman correlations. (C) An example of parasite–parasite co‐occurrence among lakes using *Unionidae* and *Thersitina*. Each point is a lake population (point size scaled by sample size), and the trend line is a Poisson general linear model curve fit with a one standard error confidence interval. (D) Parasite species co‐occurrence across host populations. Red denotes positive, and blue negative, Spearman rank correlations between parasite taxa, using host population (lake) as the level of replication. Asterisks indicate statistically significant correlations (*P* < 0.05 above diagonal, Bonferroni‐corrected below diagonal). Gray cells entail rare parasite combinations that are omitted from a given scale of the analysis. [Color figure can be viewed at wileyonlinelibrary.com]

We found evidence for significant parasite co‐occurrence at the scale of individual fish within a given lake, but these co‐occurrence patterns did not repeat themselves from one lake to the next. For instance, in McCreight Lake, 8 out of 91 pairwise comparisons between parasite taxa were significantly positively correlated (Fig. 3B), such as the abundance of *Thersitina* and Unionidae (Fig. 3A), which are both horizontally transmitted external parasites inhabiting sticklebacks’ gills. Between‐parasite correlations ranged from a lower 2.5% quantile of *r* = −0.191 to an upper 97.5% quantile of *r* = 0.368 (Appendix S1: Fig. S2A). The average Spearman correlation between any two parasites within a lake was only 0.022, but there was a systematic bias towards positive correlations (95% CI: 0.017–0.027). Although most populations had multiple significant, positive pairwise correlations (median = 8 different parasite pairs, Fig. 3B and Appendix S1: Fig. S3), there was wide variation between populations in the number and strength of these correlations, and which parasite pairs were correlated. The average (absolute) correlation strength ranged from as little as 0.068 in Upper Campbell Lake, to 0.229 in Snow Lake (ANOVA population effect *F*_32,&hairsp;3087_ = 4.74, *P* < 0.0001; Appendix S1: Fig. S2B). To illustrate variation in the number of correlations, we found 41 significant parasite–parasite correlations out of 220 pairwise comparisons in Roberts Lake (~70% were positive; Appendix S1: Fig. S3), whereas in Cecil Lake (just 200 m upstream from Roberts Lake) we observed no significant pairwise correlations among its eight parasite taxa (24 pairwise comparisons).

The strength and identity of parasite co‐occurrence differed between stickleback populations (Appendix S1: Fig. S4). The average Mantel correlation between two lakes’ co‐occurrence matrices was only *r*_M_ = 0.028. Of 526 between‐lake comparisons (excluding pairs that shared fewer than three parasite taxa), 17% exhibited significant positive correlations (6% were significantly negative). Conversely, the *testCov* analysis rejected the null hypothesis of similar co‐occurrence for most (364) pairwise comparisons between lakes (for 162 lake pairs we failed to reject the null hypothesis of similar covariance; Data S2: Supplemental_File1.csv). These among‐lake differences reflect the fact that often two parasites were correlated in some lakes, but not in other lakes where they were nevertheless both present. For example, *Dermocystidium* and *Thersitina* were positively correlated in four lakes (three different watersheds) but not in 10 other lakes where they were both present (Appendix S1: Fig. S4). As a more extreme case, some parasites (e.g., *Thersitina* and Unionidae) were significantly positively correlated in certain lakes (Fig. 3A; e.g., McCreight Lake) and significantly negatively in others (Appendix S1: Fig. S4), resulting in a nonsignificant positive relationship between Unionidae and *Thersitina* at the among populations scale (Fig. 3C), indicating that processes driving co‐occurrence at the host‐individual scale are inconsistent across the metacommunity.

It is widely noted that larger hosts harbor more parasites, which can generate positive co‐occurrences. Using general mixed models we tested whether each parasite still covaried with each other parasite after controlling for log host mass (and random intercept and slope effects of lake). As detailed in Appendix S1: Fig. S5A, even after controlling for host mass approximately a third of parasite‐parasite comparisons remain significantly associated (after conservative Bonferroni multiple test corrections). Moreover, we confirmed that for many (36%) of these associations the relationship between parasite taxa still differs in strength or direction among lakes (Appendix S1: Fig. S5B).

### Analysis 2: Parasites tend to co‐occur among host populations

**Fig. 4:**
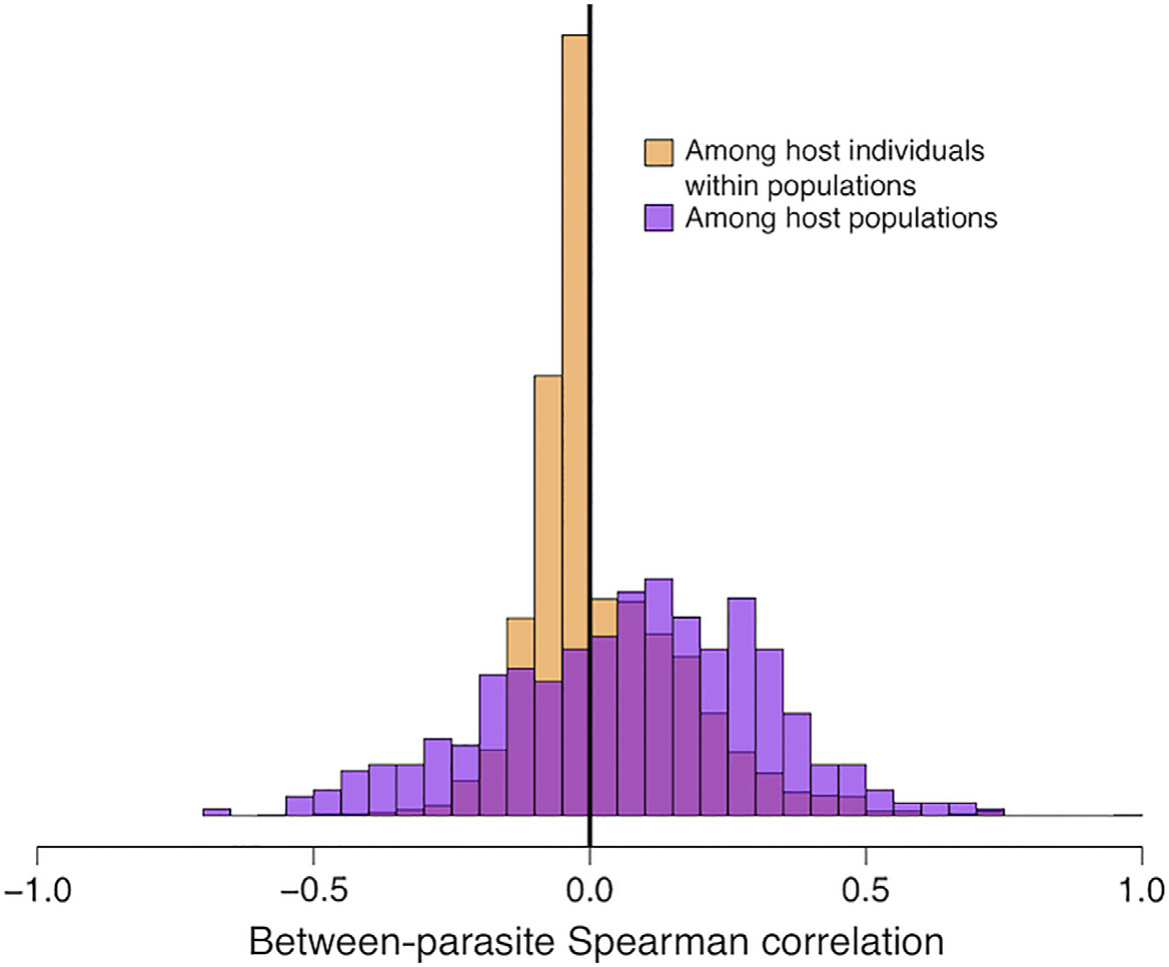
Comparison of the distribution of parasite–parasite co‐occurrences at two spatial scales. Infection intensities are more strongly correlated (positive and negative) at the scale of among‐host‐population comparisons (purple) than at the scale of variation among host individuals within populations (light orange). [Color figure can be viewed at wileyonlinelibrary.com]

We found strong co‐occurrence of parasite mean abundance at the scale of host populations (e.g., Fig. 3D). For example, *Crepidostomum* (an internal helminth with a complex multihost life cycle) and Unionidae (a directly transmitted mollusk gill parasite) tend to be either both common or both relatively rare in populations (Appendix S1: Fig. S6). Notably, among‐lake co‐occurrence involves stronger and more significant correlations than we observed within any single lake (Fig. 4).

The among‐lake co‐occurrence matrix was not related to the within‐lake co‐occurrence matrices. The Mantel correlations between these spatial scales’ matrices ranged from −0.39 to 0.34 (depending on the focal lake for individual‐scale co‐occurrence), and on average were indistinguishable from zero (mean = −0.046, SD = 0.25). These Mantel tests found no evidence that within‐ and among‐lake co‐occurrence matrices are similar. Conversely, the *testCov* analysis confirmed that all within‐lake co‐occurrence matrices were significantly different from the meta‐population wide co‐occurrence matrix (all *P* < 0.05; Data S2: Supplemental_File1.csv). These results imply that between‐parasite co‐occurrence is a result of nonrandom community assembly processes at both spatial scales, but these processes differ between lakes, and across spatial scales (within vs. among lakes).

### Analysis 3: Individual host traits predict parasite community structure within hosts

**Fig. 5:**
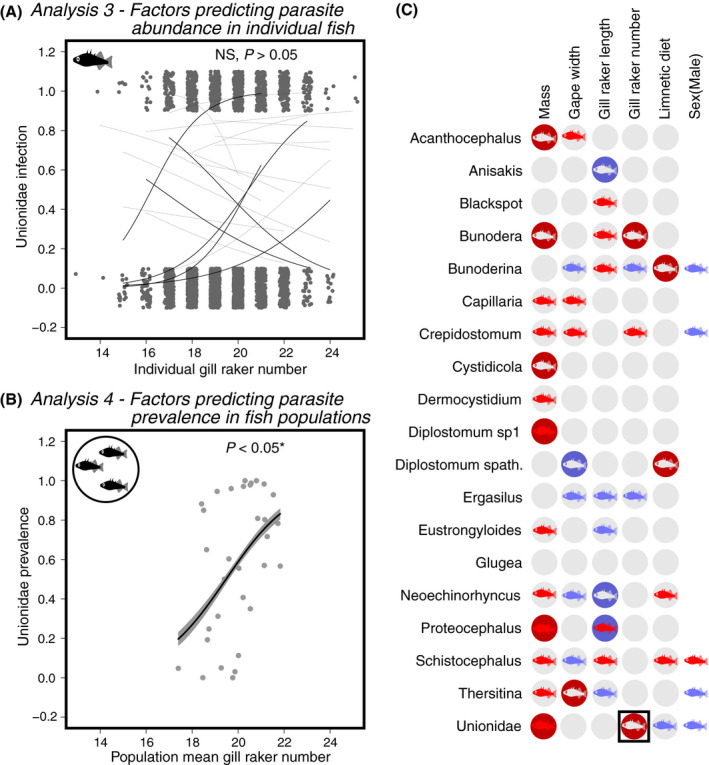
Factors that predict parasite abundance in individual hosts and host populations. (A) Comparisons of gill raker number on Unionidae infection prevalences at the scale of host individuals and (B) host populations. For host individual plots, we plot the general linear model estimate trend line for each lake with sufficient numbers of infected fish (>5); solid lines denote significant within‐lake trends, dotted thin lines are nonsignificant. The among‐individual effect size slope and standard error come from a binomial general linear model with lake as a random effect. For the among‐lake trend (B) we plot the trend line and 95% confidence interval. (C) A visual summary of scale‐dependent associations between parasite taxa and host or lake traits. Each parasite taxon (row) is compared to traits that are relevant across both scales, or uniquely relevant to within‐ or between‐population scales. For each comparison we indicate whether the host traits had a positive (red), negative (blue), or no significant effect (white, *P* > 0.01) on the given parasite, at the among‐individual scale (fish symbols), or among‐lake scale (circles). Statistical details underlying these effects are reported in Appendix S1: Tables S2 and S3. The parasite taxa in (C) are ordered on the basis of a clustering dendrogram (not shown) based on similarity in effects. [Color figure can be viewed at wileyonlinelibrary.com]

Ecomorphological characteristics explained variation in parasite abundance among individual hosts for many parasite taxa (see Appendix S1: Table S2 for statistical details). The strongest trend was for parasites to be more abundant in larger fish (Fig. 5C; Appendix S1: Fig. S7A), as has been found in other host species (Timi and Poulin 2003). This trend might reflect age, higher feeding rates, or a particular diet (larger stickleback eat more benthic prey). As noted above, the positive effect of host size on infection load of many parasites is not sufficient to explain between‐parasite co‐occurrence (Appendix S1: Fig. S5A), and the host size effect on infections differs among host populations (Appendix S1: Fig. S5B).

Associations between parasites and other trophic traits suggest that fish diet affects individual parasite loads: individuals with larger gapes (a benthic trait) had more abundant *Capillaria* and *Crepidostomum*, but fewer *Ergasilus*, *Neoechinorhyncus* and *Schistocephalus*. More numerous gill rakers (a limnetic trait) coincided with more *Crepidostomum* but fewer *Ergasilus* (Fig. 5C). Longer gill rakers (also a limnetic trait) conferred more *Blackspot*, *Bunoderina*, *Proteocephalus*, and *Schistocephalus*, but fewer *Ergasilus*, *Eustrongylides*, and Unionidae (Fig. 5C).

Stomach contents were also associated with infection. For example, individual stickleback with more limnetic diets (higher diet NMDS1 scores) had more copepod‐acquired *Schistocephalus*, but fewer *Eustrongylides* (Fig. 5C). Both trends are consistent with these parasites’ limnetic and benthic first hosts, respectively, and corroborate a prior study (Stutz et al. 2014). Host sex affected infection rates for several parasites (Appendix S1: Table S2), typically with higher infection rates in females, who also had higher parasite richness (Bolnick et al. 2020). *Schistocephalus* was the sole species that was significantly more common in males than in females, consistent with males’ tendency towards a more limnetic diet (Fig. 5C; Reimchen and Nosil 2001, Snowberg et al. 2015).

Our individual‐level GWAS analysis found no SNPs that correlated significantly with individual‐level infection after controlling for population‐level variation in both allele frequency and infection. Such GWAS analyses assume that the same loci contribute to the same adaptations in all affected populations (e.g., parallel evolution), and will fail to identify loci when different genes contribute to similar adaptations in different populations.

### Analysis 4: Host‐population traits predict parasite community structure within lakes

Variance partitioning analysis revealed that spatial distance, environment, genetic distance, and host ecomorphology each explained a significant portion (*P* < 0.05) of among‐population variation in parasite community composition. Host ecomorphology explained the most variation, and spatial distance the least (Appendix S1: Fig. S8).

**Fig. 6:**
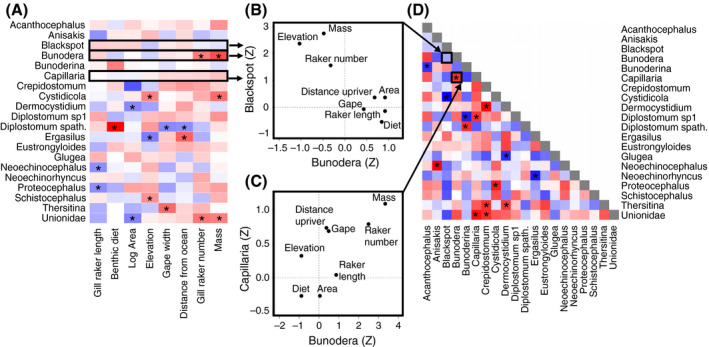
(A) Heat map of parasite dependence on host‐population characteristics, for the most common parasites. Red denotes positive correlations, blue negative. Asterisks show significant associations. (B) The negatively correlated effects (*Z* statistics from the GLM) of host‐population characteristics on two parasite taxa, Bunodera and Blackspot. Each point is a population trait, the points’ positions indicate their effect sizes on each parasite. (C) An example of positive codependence, between the parasites *Bunodera* and *Capillaria* at the scale of among‐lake variation. (D) Codependence matrix—the correlation between parasite species’ associations with environmental and host‐population traits (e.g., summarizing all pairwise comparisons as in panel (B) and (C). We calculated correlation coefficients between *Z* scores from each factor in the linear models for each species. [Color figure can be viewed at wileyonlinelibrary.com]

For individual parasite taxa, lake biogeography strongly affected the distribution of multiple parasite species across populations (Appendix S1: Table S3). Larger lakes had fewer Unionidae, *Crepidostomum* (Appendix S1: Fig. S9A), and *Dermocystidium*. Higher‐elevation lakes had higher prevalences of *Dermocystidium*, *Schistocephalus* (Appendix S1: Fig. S9B), *Diplostomum*, and *Cystidicola*, and lower prevalence of *Ergasilus* (Appendix S1: Table S2). Lakes farther up‐river from the ocean had more *Crepidostomum*, *Dermocystidium*, and *Ergasilus* but fewer *Diplostomum,* andtended to have higher infection by most parasites (mean *Z* score across all parasites = 1.95, *P* = 0.066). These effects are summarized with a heat map in Fig. 6A.

Populations with larger fish (higher mean mass) had a higher prevalence of Unionidae (Appendix S1: Fig. S7B), *Diplostomum*, *Proteocephalus*, *Bunodera*, *Cystidicola*, and *Acanthocephalus* (Fig. 5C). In fact, most parasite taxa exhibit higher prevalences in lakes with larger mean body size (averaging effect sizes [*Z*] across taxa, mean *Z* = 3.12, *P* = 0.006). Only half of these positive among‐lake associations were also observed at the individual‐host level (Fig. 5C; Unionidae, *Diplostomum*, and *Proteocephalus* were positively related to individual fish mass). Fish diet and ecomorphology also influenced infection at the among‐population level: more limnetic populations (higher diet NMDS1) were more heavily infected by *Bunoderina* (Appendix S1: Fig. S9D) and *Diplostomum spathaceum* (an association not seen at the individual host scale). *Proteocephalus* was associated with gill raker length at both spatial scales, but the direction of the effect changed from positive to negative with increasing scale. Populations that on average had more gill rakers (a typical limnetic trait) had more Unionidae (Appendix S1: Fig. SB) and *Bunodera*, neither of which were associated with gill raker number at the individual host scale. Populations with larger gape widths had more *Thersitina* but fewer *Diplostomum*; whereas neither parasite was associated with gape at the individual‐host scale.

Genome‐wide association study at the among‐population scale revealed numerous significant correlations between population allele frequencies at a given SNP and parasite prevalence. We ran 1,281,483 GLM tests (for all combinations of sufficiently polymorphic SNPs and common parasites). Applying a stringent Bonferroni correction for the multiple comparisons, we located 14,832 SNP‐parasite associations, examples plotted in Appendix S1: Fig. S10. These SNPS correlated disproportionately with a few parasites (up to 1,085 SNPs for a single parasite taxon), whereas other parasites had no significant associations. This result indicates that host genetic variation is associated with among‐host‐population variation in infection rates of some but not all parasites. Note that genetic variation associated with infection might entail immune genes, but might instead arise via genetic control of traits affecting exposure risk.

### Analysis 5: Parasite taxa exhibit co‐dependence on host‐population traits

Some groups of parasite taxa depended on the same sets of host‐population traits in Analysis 4 (Fig. 6A). For example, *Capillaria* and *Bunodera* were both more common in lakes where fish were larger and had more gill rakers, and less common in lakes with a more limnetic diet. The effects of host‐population traits on these two parasites were highly correlated (*r* = 0.722, *P* = 0.042, Fig. 6C). Conversely, we observe negative co‐dependence such as *Cystidicola* and Blackspot which respond to similar host traits but in opposite directions (*r* = −0.913, *P* = 0.002). Other pairs of parasites exhibited no significant co‐dependence, such as Blackspot and *Bunodera* (Fig. 6B). These positive and negative “co‐dependences” of various combinations of parasite taxa are summarized in Fig. 6D.

### Comparing results across spatial scales

The preceding analyses documented significant associations between parasite taxa, and between parasites and host or lake traits. Many of these associations were only observed at one spatial scale: among host individuals (Appendix S1: Table S2), or among host populations (Appendix S1: Table S3), but not both, as summarized graphically in Fig. 5C. We already explicitly compared scales by statistically confirming that parasite–parasite co‐occurrences were stronger (positive and negative) at the among‐lake scale (Fig. 4). Here, we quantitatively show that associations between parasites and host (or population) traits differ across scales. First, we confirmed that there exist parasite‐trait correlations that act in the same direction at both spatial scales, such as the positive effect of host mass on Unionidae abundance and prevalence (Appendix S1: Fig. S7A, B; Fig. 5C). However, even when the effect direction is consistent across scales, in all such cases the effect‐size estimates had nonoverlapping confidence intervals. Second, we observe numerous cases where a host trait affects parasite infection status exclusively among host individuals (e.g., *Bunoderina* and gill raker number, Appendix S1: Fig. S7E, F), or exclusively among host populations (e.g., mean gill raker number on Unionidae infection; Fig. 5A, B). In such cases, it is not simply that we lack power at one spatial scale. Rather, we confirm that effect size estimates are stronger at one scale than another (nonoverlapping confidence intervals). As an extreme example, gill raker length had significant effects on *Proteocephalus* at both scales, but in opposing directions (Fig. 5C).

## Discussion

Parasites form hierarchically structured metacommunities, whose composition varies among host individuals within populations, and between host populations (Combes 2001, Poulin 2007, Mihaljevic 2012, Seabloom et al. 2015, Borer et al. 2016). Past studies of snails’ trematode parasites, and *Daphnia*’s pathogens, among others, have documented scale‐dependent factors governing these parasite metacommunities’ composition (Seabloom et al. 2015). Here, we contribute a detailed example of scale dependence in a metacommunity of stickleback parasites, evaluating effects of abiotic environment, host sex, size, morphology, diet, and genomic variation. Our analysis suggests that most variables governing this metacommunity act either at the among‐individual scale, or the among‐population scale, but rarely at both scales (host body size being the lone exception).

The stickleback parasite metacommunity examined here is moderately deterministic: some combinations of species tend to co‐occur more often than expected by chance. At the scale of co‐occurrence within host individuals in a given lake, we observe mostly sparse cases of positive correlations. These positive correlations are partly but not fully explained by shared associations with host body size, and are inconsistent from one lake to the next. Parasite co‐occurrence is more common and stronger at larger spatial scales (among host populations). At this larger scale we see more equal numbers of positive and negative associations. These among‐population patterns of co‐occurrence are different in strength, direction, and parasite combinations, when compared to within‐population trends.

Previous studies have also found scale‐dependent co‐occurrence. For instance, for trematodes in California horn snails (Kuris and Lafferty 1994) co‐occurrence tends to be negative at the level of individual hosts (whereas we see mostly positive associations), but strongly positive among host populations (whereas we see a mix of strong positive and negative associations). The implication is that, as one moves to the larger spatial scale, the parasite community becomes more deterministically structured by habitat patch characteristics (lake features or host‐population‐trait means). Consistent with this inference, parasite variation among host populations is more strongly regulated, by a greater number of biotic and abiotic factors, than variation among host individuals.

### Spatial scale and the factors affecting parasite community structure

Parasite metacommunity structure can broadly be explained by a combination of dispersal and persistence filters, equivalent to what parasitologists call encounter and compatibility filters, respectively (Fig. 1). We found that parasite communities depended on three interrelated ecological encounter filters: host diet, trophic morphology, and size. In a few cases these ecological filters acted at both spatial scales. For example, *Diplostomum* was more common in larger host individuals and in larger (on average) host populations, a trend found in 3 of the 21 parasite taxa examined. This trend is consistent with body‐size effects seen in many other fish species (Timi and Poulin 2003), perhaps reflecting greater age and greater time to accrue parasites. Host body size was the only trait to exhibit consistent effects on infection at both scales frequently.

*Proteocephalus* also covaried with an ecological filter (gill raker length) at both spatial scales, but the sign of this correlation was reversed. More typically, however, host diet and morphology had effects on parasite abundance at one scale, but not another. This scale dependence is counter to our expectation that trophically transmitted parasites should exhibit similar ecological encounter filters at both spatial scales. This surprising result might be due to scale‐specific effects of other filters (e.g., immunity) that counteract the ecological filters at one scale but not another. A particularly important filter, for which we lacked data, is the distribution of parasites’ terminal hosts (e.g., piscivorous birds for *S. solidus* cestodes). Variation in terminal host abundance, feeding behavior, and migratory flyways could be a major force dictating parasite distributions among lakes, overriding effects of host ecology that come into play within lakes where a parasite is present.

Some filter mechanisms are only applicable at one spatial scale. For instance, males and females differed in parasite infections, consistent with previous studies in stickleback (Reimchen and Nosil 2001). Because our populations do not differ greatly in sex ratio, sexual dimorphism should primarily contribute to within‐population metacommunity variation. Conversely, geographical characteristics of entire lakes (size, elevation, distance from ocean) are necessarily shared by all individuals within a given lake and so only contributed appreciable variation at the among‐population scale. Another exclusively large‐scale consideration is geographic distance between populations, which modifies rates and sources of parasite colonization. Our variance decomposition analysis of metacommunity composition found no effect of between‐lake distances overland (“as the crow flies”), which is a potentially relevant metric for stickleback parasites that have birds as terminal hosts. In contrast, this analysis did support a small effect of distance along waterways (“as the fish swims”), controlling for the effects of between‐population relatedness, as interconnected lakes within a watershed tend to be genetically more similar (Caldera and Bolnick 2008). We conclude that stickleback parasite communities within lakes are (at the scale of tens of kilometers) not substantially dispersal limited.

### Coevolution

The variance partitioning analysis confirms that genetically divergent host populations tend to have more divergent parasite communities, controlling for the relatively weak confounding effect of spatial distance. This result suggests that there is genetic variation in infection risk (because of heritable exposure or resistance), which arises in large part from shared ancestry (affecting whole‐genome *F*_ST_), not just from selection on particular loci. This result is consistent with a previous study in Scottish lakes that also showed that parasite community composition was more similar between genetically similar lakes of stickleback (Rahn et al. 2016). But, evolved differences in host resistance (Weber et al. 2017) can also contribute to parasite metacommunity structure.

Host and parasite (co)evolution are most relevant to population‐scale metacommunity structure, because natural selection acts on populations, not individuals. In fact, selection should have opposing effects on metacommunity variation at these scales. Selective sweeps within host populations simultaneously increase between‐population differences and reduce within‐population polymorphism (reducing among‐individual genetic variation in resistance). Such sweeps will generate genotype–parasite associations at the among‐population level, but remove variance to detect such effects within populations. Alternatively, parasites might impose balancing selection within host populations (Wegner et al. 2003), promoting among‐individual associations between genotype and resistance, but inhibiting population divergence. Our genome‐wide association (GWAS) analyses found numerous SNPs associated with infection variation among populations, but not among individuals within populations. This scale‐dependent effect of particular loci suggests that, in this system, parasites primarily drive divergent selection between populations, rather than balancing selection within populations.

As noted in the introduction, hosts can evolve resistance to severe and locally common parasites. Over evolutionary time this leads to a decoupling, or even reversal, of the relationship between ecological exposure risk vs. observed infection rate (Fleischer et al. 2020). For instance, some lake populations of stickleback have evolved particularly effective resistance to *S. solidus* cestodes (Weber et al. 2017). This population‐level evolution of resistance means that lake populations with high intake of copepods, the intermediate host of *S. solidus*, are not more infected by this parasite (Stutz et al. 2014). We therefore posit that the evolution of immunity among populations, confirmed by our GWAS analysis, might explain why diet and trophic morphology effects that we observed within host populations (Appendix S1: Table S2) were not repeated at the among‐population scale (Appendix S1: Table S3).

### Codependence and co‐occurrence

Most parasite taxa in this metacommunity are regulated by a combination of host traits at the individual‐host scale, and at the between‐host‐population scale are regulated by abiotic conditions, host traits, and host allele frequencies. At the population scale, some combinations of parasites respond in the same directions to the same sets of population traits (e.g., show co‐dependency). Other combinations of parasite taxa respond in opposite directions or are simply independent (Fig. 6). These patterns of shared (or opposing) dependence on lake and host traits likely represent a mechanistic explanation for the positive and negative co‐occurrence documented above (Fig. 3D).

## Summary

The stickleback parasite metacommunity studied here is structured by a wide variety of factors: lake geography, host mean traits, host allele frequencies, and individual traits (including sex). These effects are almost all scale‐dependent (except host body size), which means that the mechanistic basis of infection and epidemiology cannot readily be generalized from individual animals to their populations, or vice versa. A similar message arises from considering parasite diversity, rather than identity: the number of parasite taxa per fish depends on host and population traits, but these effects differ between individual‐ and host‐population scales (Bolnick et al. 2020). Together, these analyses illustrate the general point that filters structuring metacommunities are highly scale‐dependent. Such scale‐dependent effects can explain inconsistent findings among studies conducted at different scales, and imply that multiscale studies should be the norm for parasite metacommunity ecology. Importantly, our results also highlight the potential for host evolution (as revealed by our GWAS analyses) to override and obscure ecologically driven associations between host traits, risk, and actual infection.

## Supporting information

Appendix S1

Data S1

Data S2

Metadata S1

Metadata S2
