# Supplementary material for: Scale‐dependent effects of host patch traits on species composition in a stickleback parasite metacommunity
Source: Ecology. 2020 Oct 1;101(12):e03181. doi: 10.1002/ecy.3181 (PMC7757261; doi:10.1002/ecy.3181)
Supplement: Supplementary file 1 — Appendix S1 [file ECY-101-e03181-s001.pdf]

Supporting Information. Bolnick, D.I., E.J. Resetarits, K. Ballare, Y.E. Stuart, and W.E. Stutz. 2020. Scale-dependent effects of host patch traits on species composition in a stickleback parasite metacommunity. Ecology.

Appendix S1

Figure S1. A) Vancouver Island, with a black dot indicating the location of the inset map (B), where lake, stream, and marine sample sites are indicated in blue, green, and black points.

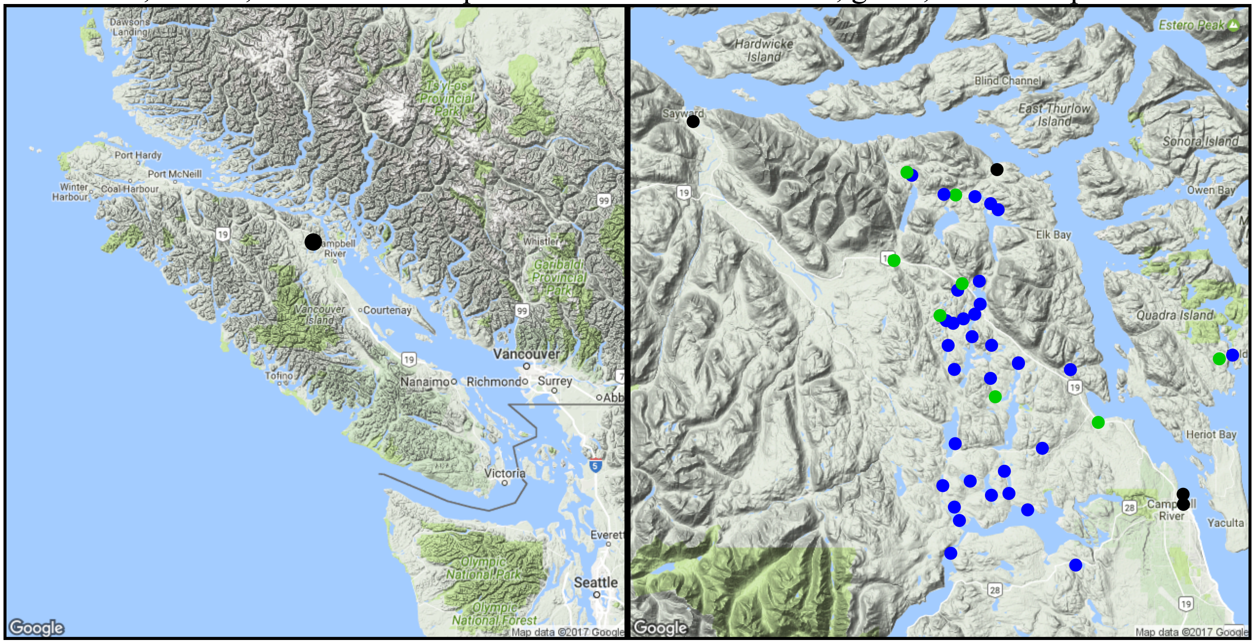

Figure S2. A) The distribution of within-lake between-parasite Spearman rank correlations, for all comparisons within all lakes. Note the two modes, positive and negative. B) The distribution of lake-level average correlation coefficients, taking the absolute value before averaging to focus on correlation strength.

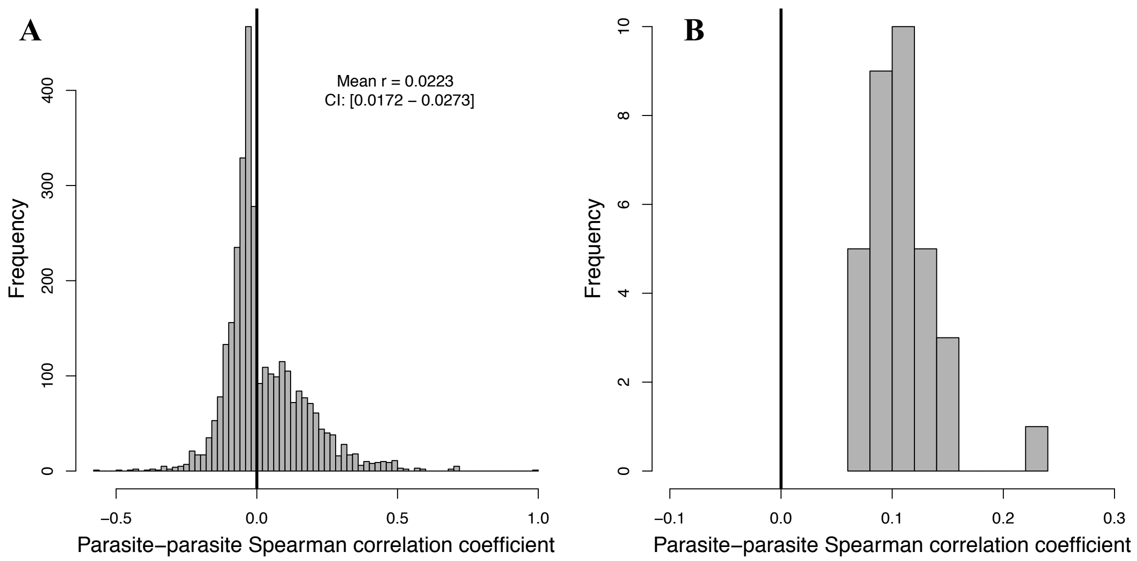

Figure S3. Co-occurrence matrix of parasites among individual stickleback in Roberts lake (N = 250 fish). Numbers represent the number of hosts carrying a given parasite.

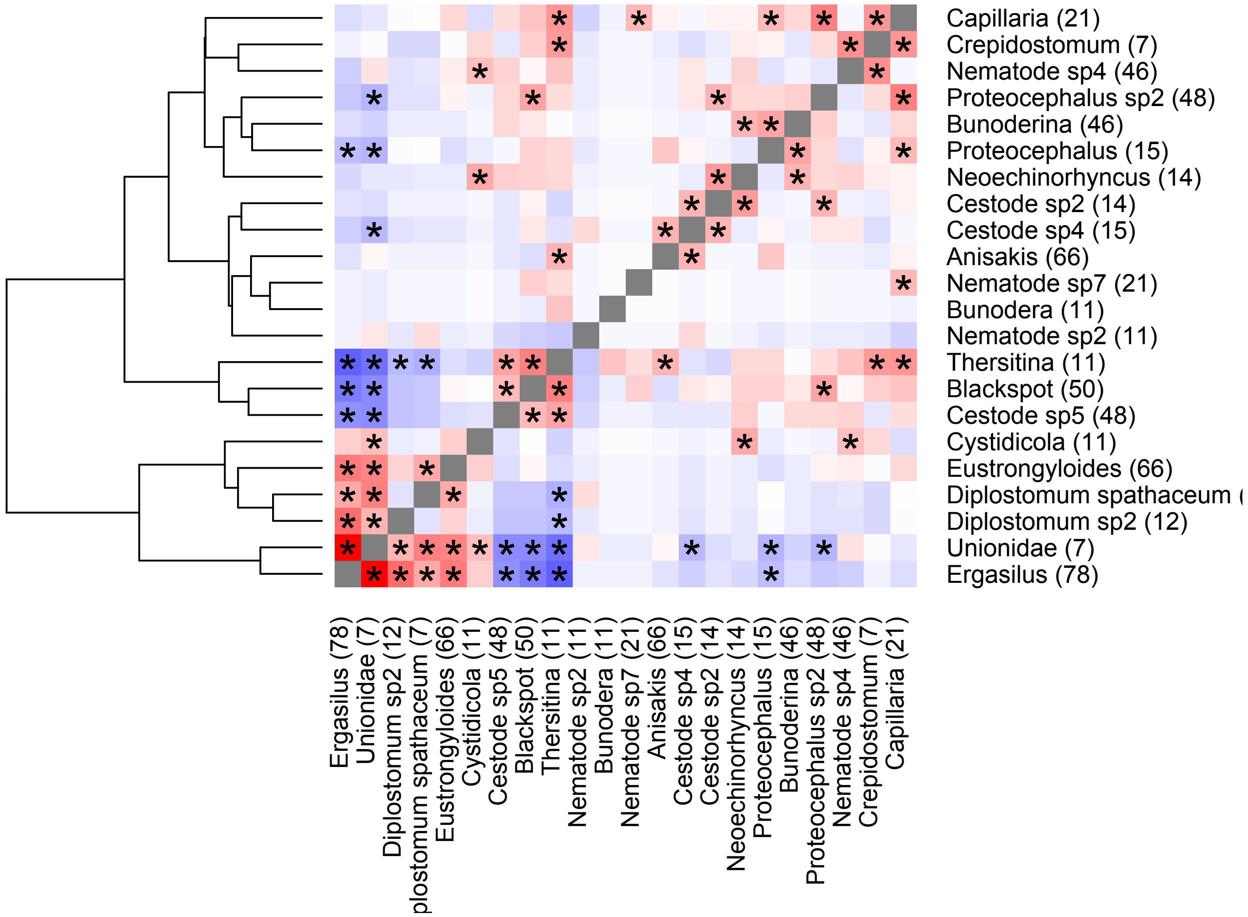

Figure S4. Parasite-parasite correlations are inconsistent among host populations. Each column is a host population, with rows representing parasite-parasite comparisons. The farthest-left column represents the among-population correlation matrix (Fig. 5), for comparison. Red and blue denote positive and negative correlations, respectively. White indicates pairwise comparisons for which one or both parasites are absent in a given lake. Asterisks indicate significant comparisons within each population after within-population FDR correction.

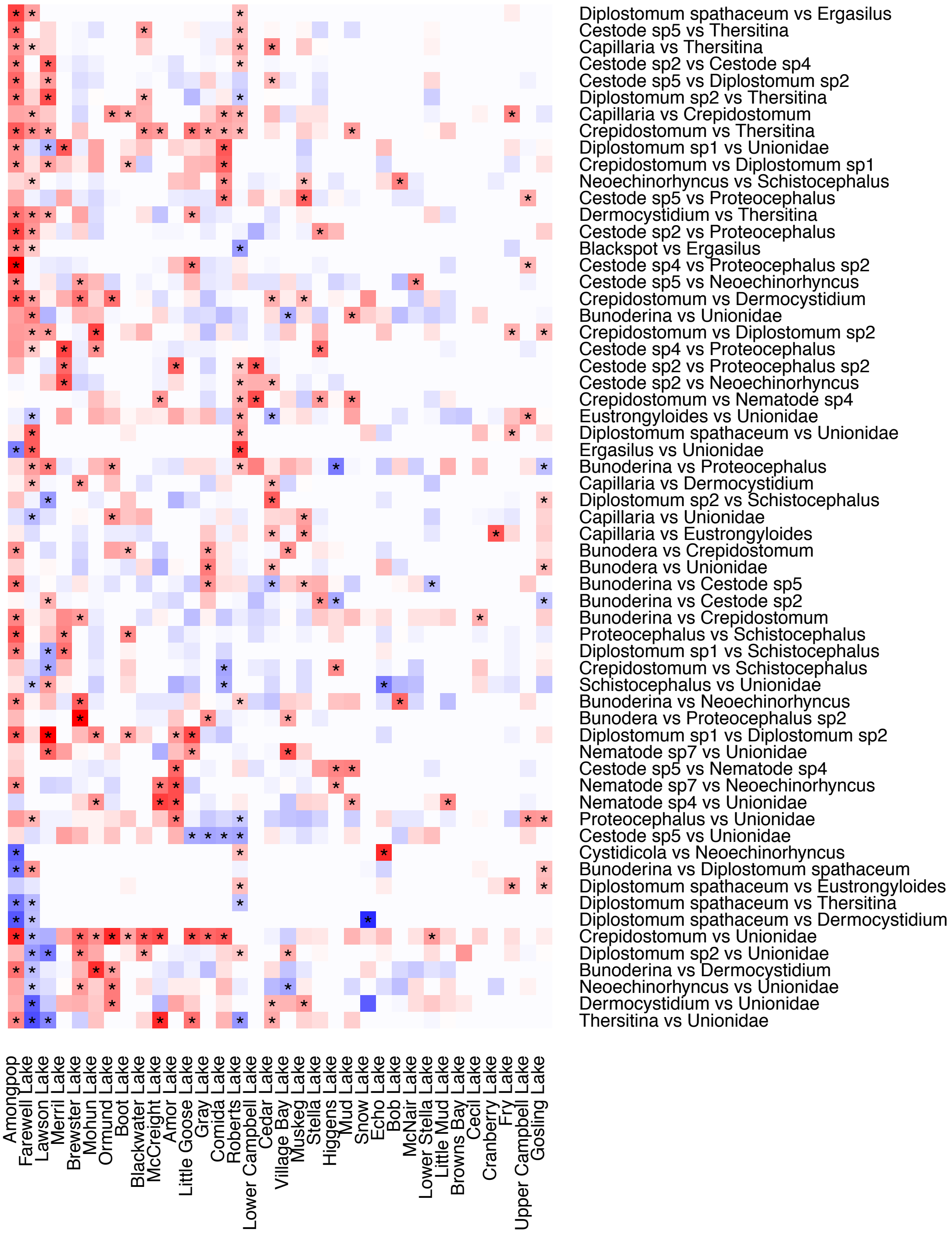

Figure S5. We re-evaluated parasite-parasite co-occurrence patterns while controlling for fish mass. To do so, for every pair of parasite taxa *i* and *j* we used a negative binomial general linear model to evaluate how parasite *i*'s abundance depends on lake (random intercept effect), host mass (fixed effect plus random among-lake slope), and the abundance of parasite *j* (fixed effect plus random among-lake slope). The two key goals here are illustrated in the two panels. (A) To confirm that parasite-parasite co-occurrence persists when controlling for their shared dependence on host mass, we highlight significant main effect co-occurrences (red) after strict Bonferoni correction. Black matrix elements are non-significant or fail to pass Bonferroni correction. White elements could not be estimated because there were too few fish or lakes in which both parasites co-occurred so the mixed model failed to converge. The final column indicates (in red) parasites whose infection intensity exhibits a main effect of host log mass. (B) To confirm that parasite-parasite co-occurrences differ among lakes, we highlight significant lake*parasite *j* interactions (random slope effects) with red matrix elements, after strict Bonferroni correction. As in (A), black elements are non-significant or fail multiple test correction, and white elements could not be estimated. The last column indicates (in red) the parasites whose infection intensity exhibits a significant lake by log host mass interaction (random slope effect).

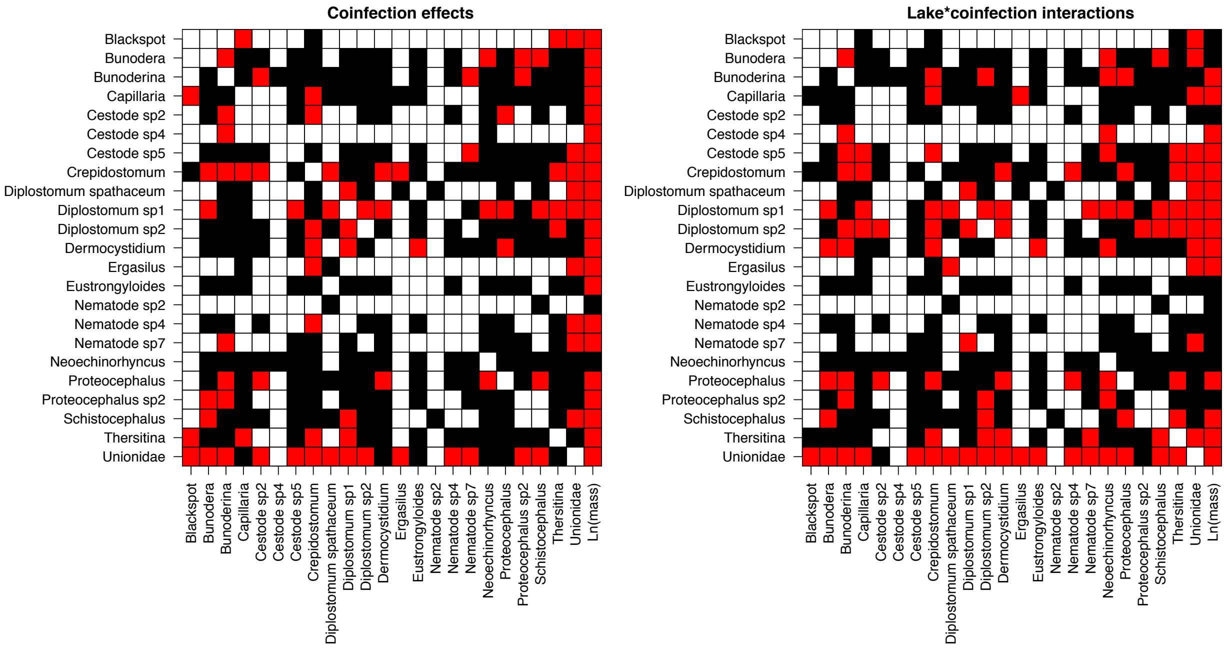

Figure S6. An example of parasite-parasite co-occurrence among lakes. Each point is a population (points scaled by their sample size), and the trendline is a Poisson general linear model curve fit with a one standard error confidence interval.

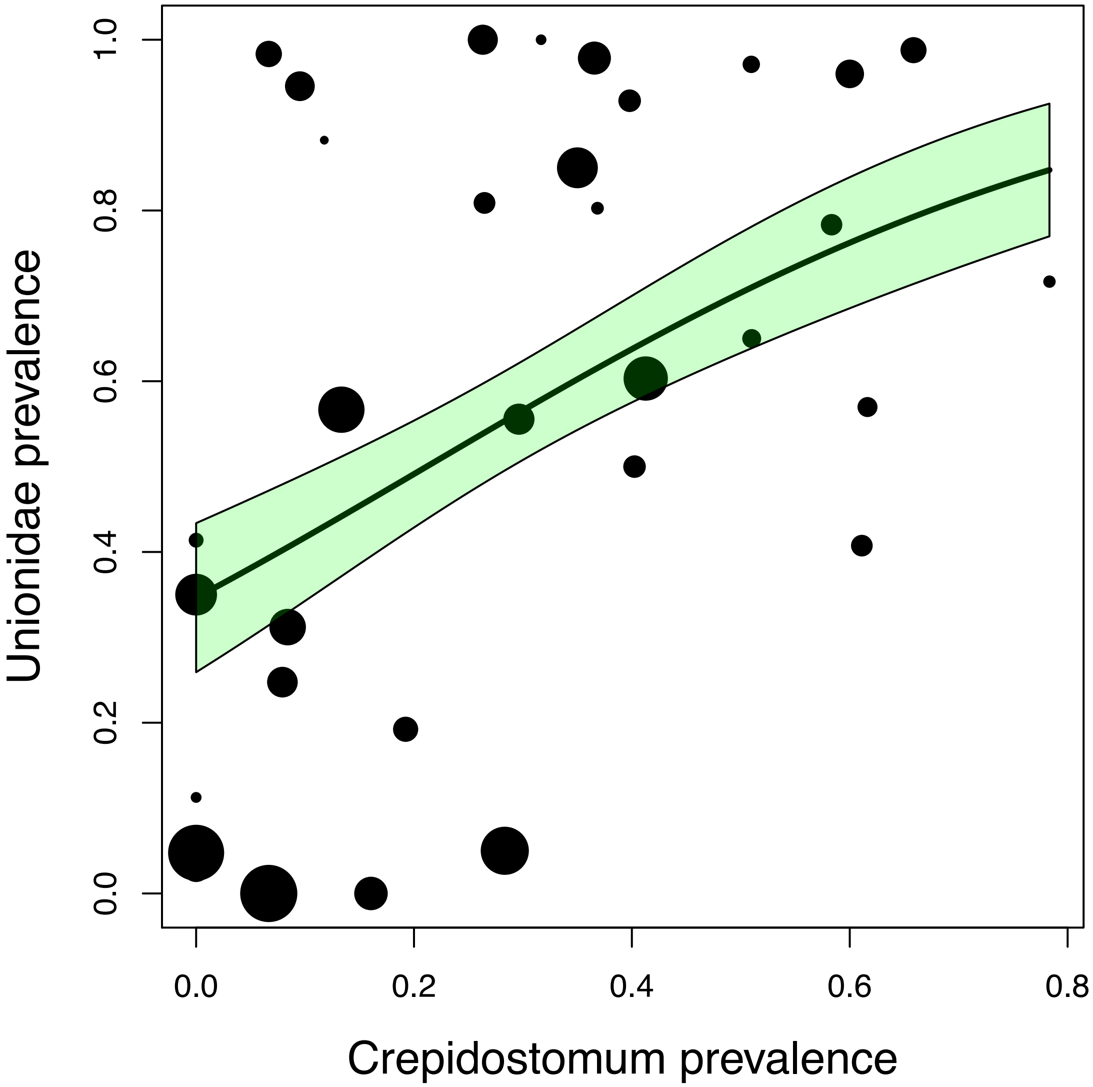

Figure S7. Comparisons of host trait effects on parasite infection prevalences at the scale of host individuals (left column, red points) and host populations (right column, blue points). For host individual plots, we plot the general linear model estimate trendline for each lake with sufficient numbers of infected fish (>5); thick solid lines denote significant within-lake trends, dotted thin lines are non-significant. The among-individual effect size slope and standard error come from a binomial general linear model with lake as a random effect. When the main effect of host traits is strong relative to among-lake variance in slopes, we also plot the main effect trend with its confidence interval. For among-lake panels we plot the trendline and 95% confidence interval. We use the same x and y axis scale for each pair of plots in a row, to facilitate visual comparison of their slopes, formalized by comparisons between their estimated slopes.

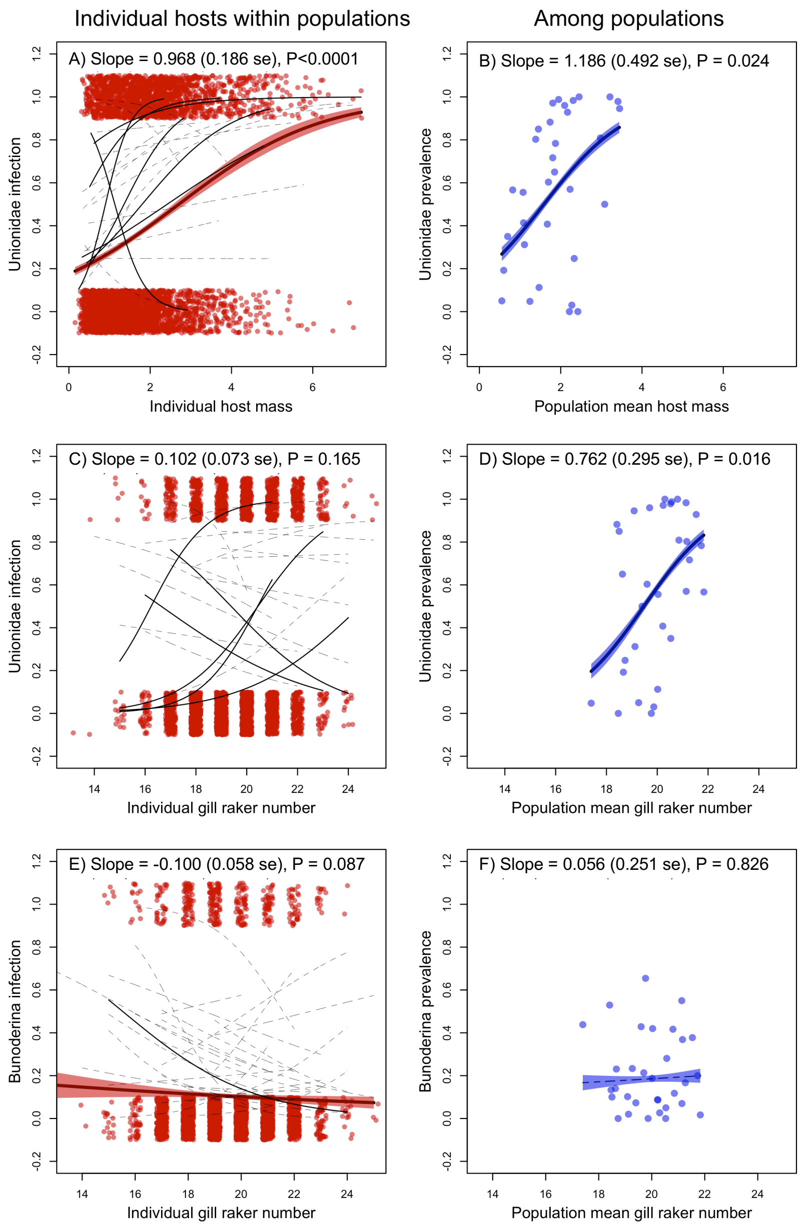

Figure S8. Variance partitioning analysis, estimating the extent to which among-lake variation in parasite community composition depends on spatial distance, genetic distance, phenotypic distance, and habitat differences. Numbers in each cell represent represent the percent variance explained by each factor or interactions among factors (at intersections).

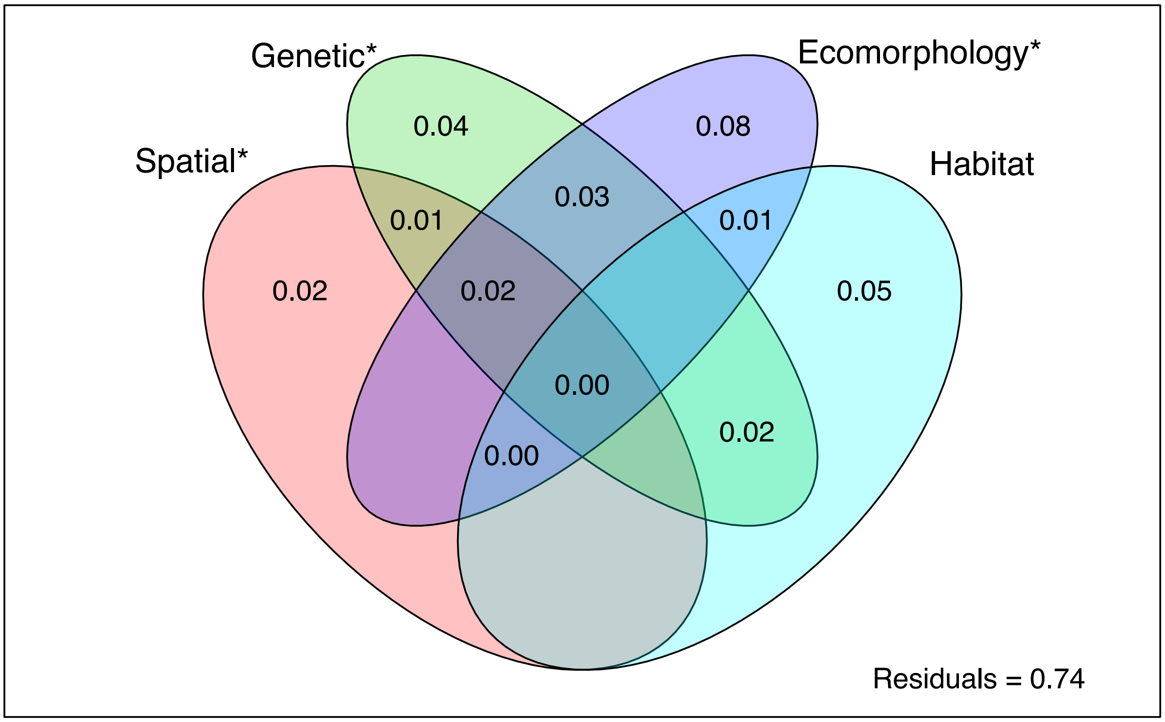

Values <0 not shown

Figure S9. Examples of associations between parasite prevalence (% hosts infected) and lake traits, for (A) lake area, (B) lake elevation. GLM trendlines with one standard error confidence estimates are plotted. Lakes are the level of replication in this figure. These associations are summarized in Table S2.

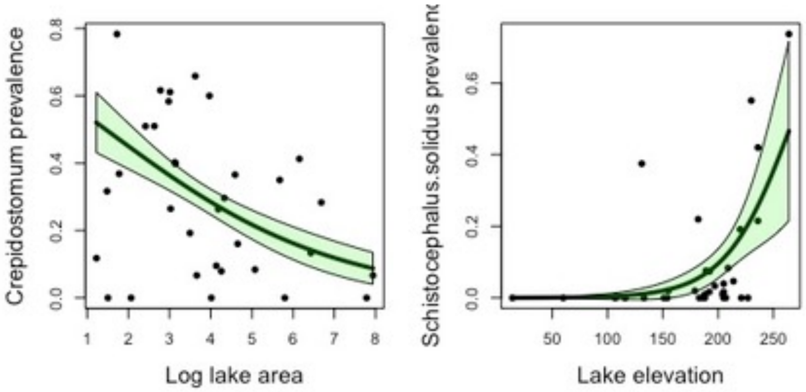

Figure S10. Example of strong GWAS results for various parasite taxa, chosen as illustrative examples. For each taxon we plot the position of GWAS associations (blue dots) along chromosomes, showing only the top 20 associations significant after Bonferroni correction. Next to each map, we plot the association between SNP frequency versus the parasite prevalence, for one of the strongest-effect SNPs for that parasite. Points represent populations, with a binomial GLM curve fit. Above each association plot we list the parasite taxon and the linkage group and base position on that chromosome.

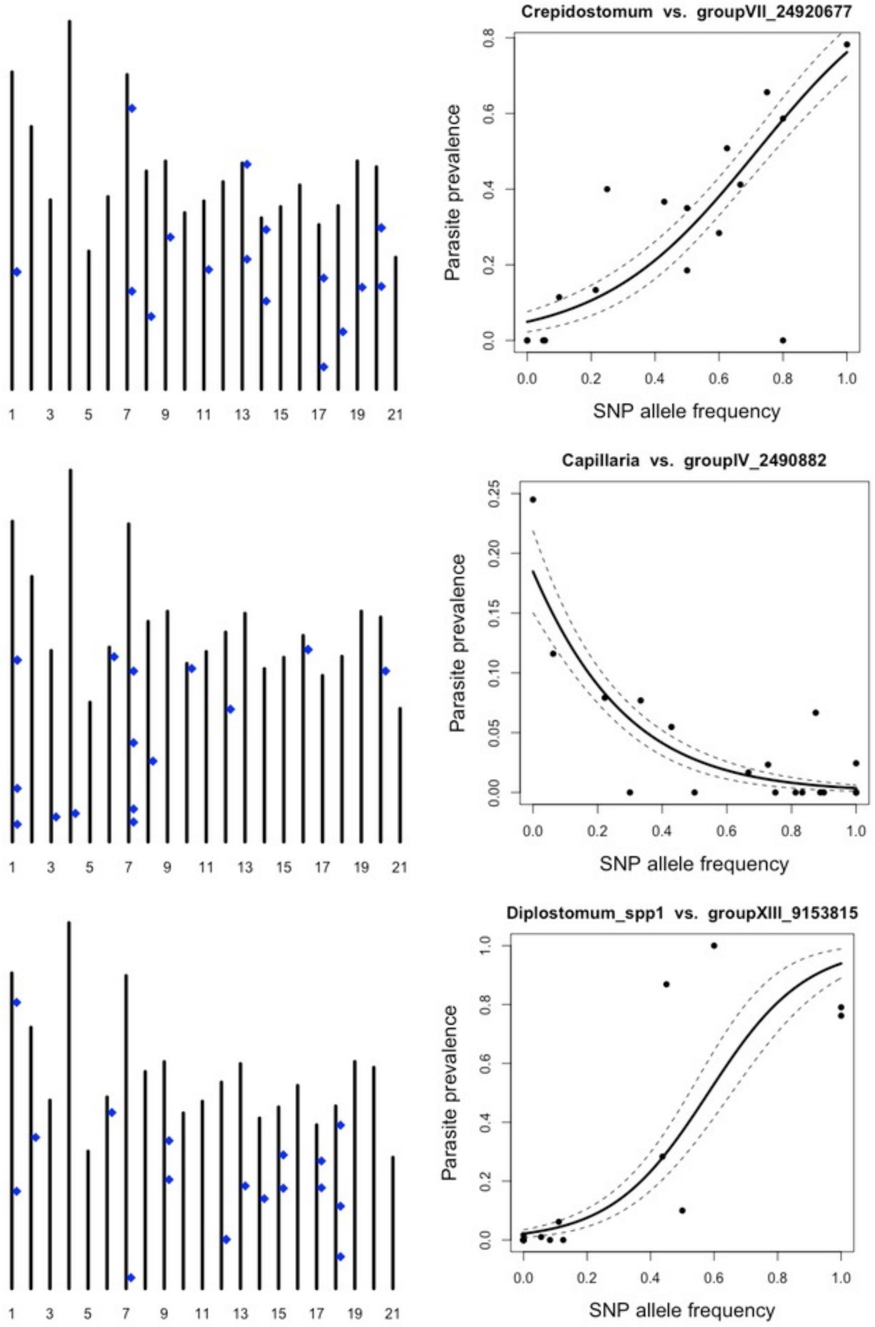

Figure S10 (continued)

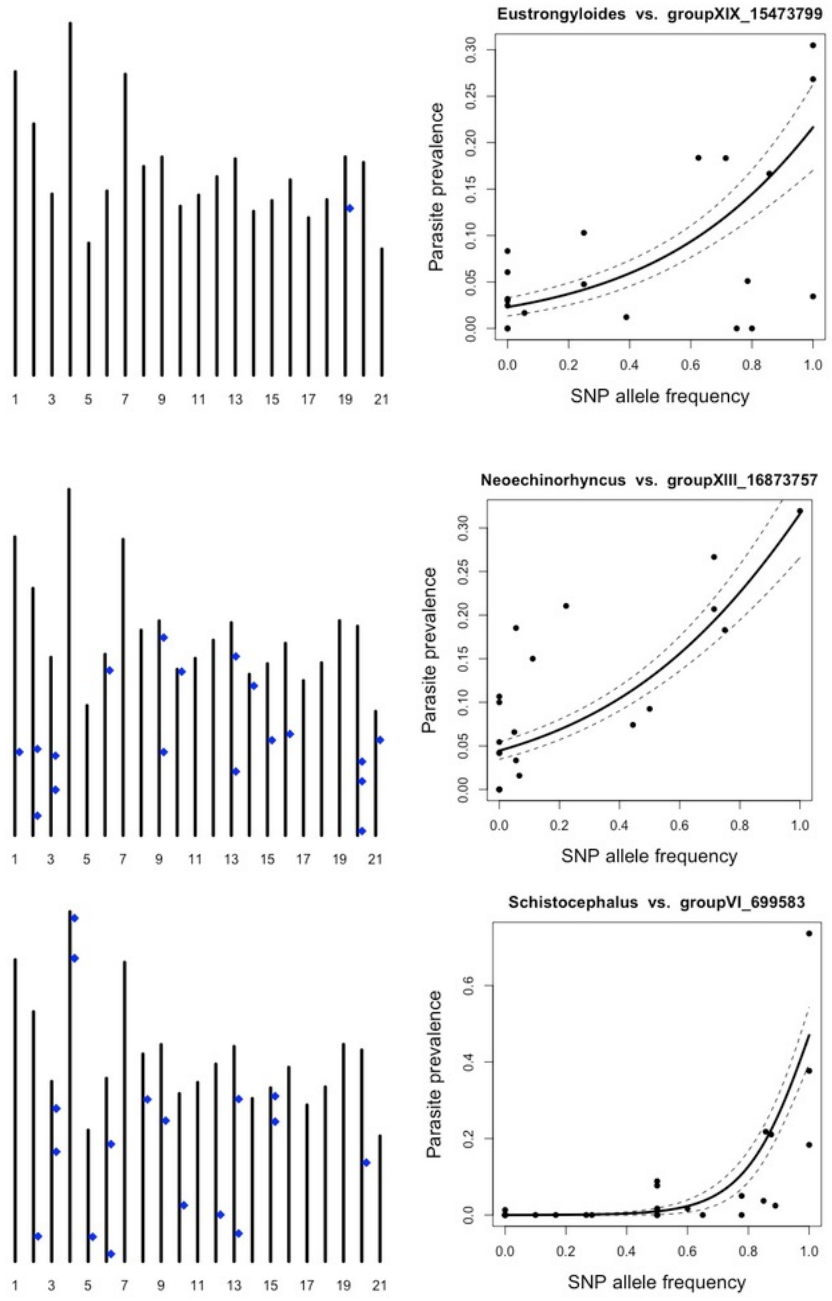

Figure S10 (continued)

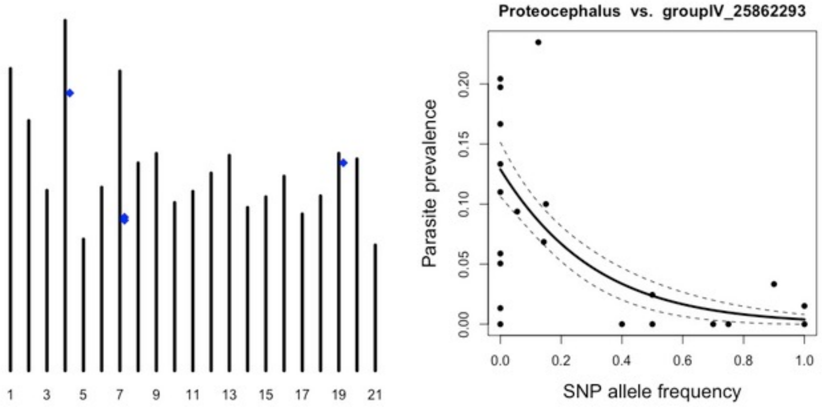

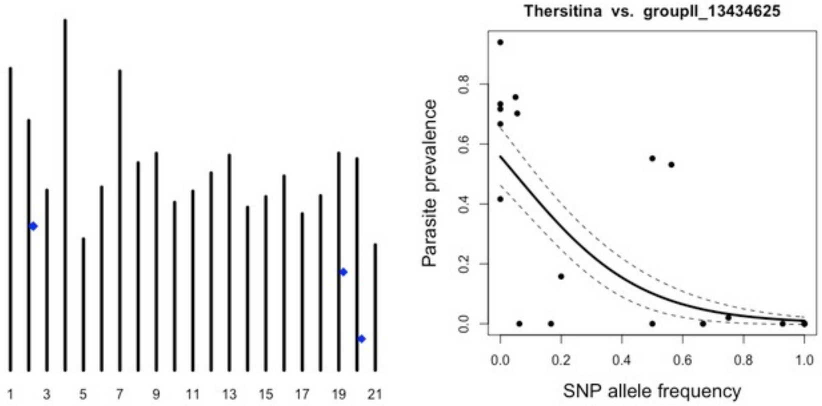

Table S1 Summary of sampled populations, locations, sample sizes, and mean parasite taxon richness.

Site name	Longitude	Latitude	Total	Morphology	Diet	ddRAD	Habitat	Watershed
Adam and Eve Estuary	-131.9426	50.4012	99	99	0	10	Estuary	Adam River
Amor Bridge	-125.6639	50.2543	60	30	23	10	Stream	Amor River
Amor Lake	-125.5791	50.1581	60	30	30	10	Lake	Amor River
Blackwater Lake	-125.5878	50.1794	82	30	25	16	Lake	Amor River
Bob Lake	-125.5281	50.3045	80	61	61	10	Lake	Pye River
Boot Lake	-125.5270	50.0467	93	93	0	0	Lake	Campbell River
Brewster Lake	-125.5779	50.0924	63	30	28	10	Lake	Campbell River
Browns Bay Lake	-125.4159	50.1580	29	0	0	10	Lake	Browns Bay Creek
Campbell River Marsh	-125.2570	50.0387	105	105	0	0	Estuary	Campbell River
Campbell River Point	-125.2574	50.0476	173	173	0	0	Estuary	Campbell River
Cecil Lake	-125.5440	50.2361	100	100	0	10	Lake	Amor River
Cedar Lake	-125.5664	50.2029	98	30	29	10	Lake	Amor River
Comida Lake	-125.5283	50.1502	86	86	0	0	Lake	Mohun River
Comida Stream	-125.5215	50.1339	192	192	0	0	Stream	Mohun River
Cranberry Lake	-125.4553	50.0883	66	66	0	10	Lake	Mohun River
Echo Lake	-125.4084	49.9848	68	68	0	10	Lake	Quinsam River
Farewell Lake	-125.5905	50.2013	216	216	0	0	Lake	Amor River
Farewell Stream	-125.5992	50.2058	260	260	0	0	Stream	Amor River
Fry Lake	-125.5720	50.0244	101	101	0	11	Lake	Campbell River

Gosling Lake	-125.5023	50.0483	147	146	0	0	Lake	Campbell River
Gray Lake	-125.5953	50.0553	75	30	27	9	Lake	Campbell River
Higgens Lake	-125.5088	50.0680	81	30	29	10	Lake	Campbell River
Lawson Lake	-125.5790	50.0362	82	30	27	10	Lake	Campbell River
Little Goose Lake	-125.4890	50.1634	76	31	28	10	Lake	Mohun River
Little Mud Lake	-125.5505	50.2069	60	30	29	10	Lake	Amor River
Lower Campbell Lake	-125.4760	50.0338	30	30	30	10	Lake	Campbell River
Lower Stella Lake	-125.5501	50.3107	104	30	30	10	Lake	Pye River
McCreight Lake	-125.6390	50.3295	60	30	28	10	Lake	Amor River
McCreight Stream	-125.6457	50.3321	60	30	29	10	Stream	Amor River
McNair Lake	-125.5744	50.2281	60	30	28	10	Lake	Amor River
Merril Lake	-125.5568	50.0593	38	30	28	10	Lake	Campbell River
Mohun Creek	-125.3767	50.1113	27	27	21	10	Stream	Mohun River
Mohun Lake	-125.4890	50.1640	60	30	28	10	Lake	Mohun River
Mud Lake	-125.5541	50.1871	60	30	28	10	Lake	Amor River
Muskeg Lake	-125.5808	50.1989	104	30	28	10	Lake	Amor River
Ormund Lake	-125.5267	50.1795	60	30	28	10	Lake	Amor River
Pye Creek	-125.5772	50.3122	60	29	27	0	Stream	Pye River
Pye Outlet	-125.5191	50.3345	73	73	0	10	Estuary	Pye River
Roberts Lake	-125.5428	50.2158	250	250	0	0	Lake	Amor River
Roberts Stream	-125.5681	50.2339	354	345	0	0	Stream	Amor River

Sayward Estuary	-125.9464	50.3769	100	100	0	10	Estuary	Salmon River
Snow Lake	-125.5937	50.3127	17	17	3	9	Lake	Pye River
Stella Lake	-125.5175	50.2992	60	30	30	11	Lake	Pye River
Upper Campbell Lake	-125.5841	49.9953	105	30	29	10	Lake	Campbell River
Village Bay Lake	-125.1878	50.1708	81	60	59	0	Lake	Village Bay
Village Bay Stream	-125.2064	50.1675	60	30	29	0	Stream	Village Bay

Table S2. General linear model results from individual-host scale regressions of parasite intensity on individual host traits. We provide slope estimates and standard errors and P-values for the main effects listed, and a P-value for the random slope effect of population. Positive and negative effects are shaded red and blue, respectively, for all significant ($P < 0.05$, also in bold font) and marginally significant ($P < 0.1$, not bold font) associations. Significant random slope effects of lake are shaded grey.

	Parasite taxon	Acanthocephanus	Blackspot	Bunodera	Bunoderina	Capillaria	Crepidostomum	Cystidicola	Diplostomum spathaceum	Diplostomum spp	Dermocystidium	Ergasilus	Eustrongyloides	Neoechinorhyncus	Neoechinocephalus	Proteocephalus	Schistocephalus	Thersitina
Sex	Slope	-0.235	-0.277	-0.257	**-0.385**	-0.109	**-0.478**	0.206	-4.424	-0.002	-0.011	-0.005	0.209	0.159	-0.695	-0.093	**0.630**	**0.587**
	Std Error	0.657	0.291	0.544	**0.193**	0.257	**0.152**	0.368	6.110	0.273	0.279	0.529	0.323	0.295	0.664	0.297	**0.237**	**0.241**
	P	0.720	0.342	0.636	**0.046**	0.670	**0.002**	0.575	0.469	0.995	0.968	0.992	0.518	0.592	0.296	0.754	**0.008**	**0.015**
	Random effect P	1.000	1.000	0.087	1.000	0.230	0.991	1.000	**0.034**	1.000	0.193	0.052	0.274	0.256	0.519	0.234	0.957	0.164
Mass	Slope	0.965	0.323	0.393	0.090	**0.745**	**0.984**	0.721	1.743	**1.025**	**0.656**	0.406	**1.036**	**1.072**	**-0.681**	**0.546**	**0.583**	**1.000**
	Std Error	0.586	0.304	0.332	0.198	**0.217**	**0.165**	0.522	6.668	**0.301**	**0.222**	0.296	**0.276**	**0.254**	**0.303**	**0.206**	**0.212**	**0.193**
	P	0.100	0.288	0.236	0.650	**0.001**	**0.000**	0.167	0.794	**0.001**	**0.003**	0.169	**0.000**	**0.000**	**0.024**	**0.008**	**0.006**	**0.000**
	Random effect P	1.000	0.958	0.186	0.992	**0.487**	**1.000**	0.998	1.000	1.000	0.428	**0.114**	0.550	**0.524**	0.812	0.493	**0.998**	0.335
Gape	Slope	5.010	1.744	0.555	-1.532	**1.944**	**2.275**	-1.744	-7.841	-0.863	0.967	**-2.661**	-0.460	**-2.465**	-2.427	-0.161	**-2.482**	0.792
	Std Error	2.617	1.268	1.333	0.879	**0.845**	**0.679**	1.482	13.784	1.220	0.962	**1.256**	1.068	**1.159**	1.457	0.888	**0.964**	0.786
	P	0.056	0.169	0.677	0.081	**0.021**	**0.001**	0.239	0.569	0.479	0.315	**0.034**	0.667	**0.033**	0.096	0.856	**0.010**	0.313
	Random effect P	1.000	1.000	0.227	1.000	0.487	1.000	1.000	1.000	1.000	0.428	0.151	0.550	0.524	0.812	0.493	0.998	0.380
Gill raker length	Slope	-1.427	**1.377**	1.935	**1.582**	0.140	-0.147	-0.880	2.273	-1.104	0.253	**-1.903**	**-1.199**	0.439	-0.645	**1.146**	**1.396**	-0.089
	Std Error	1.367	**0.588**	1.040	**0.534**	0.429	0.365	1.163	10.736	0.720	0.524	**0.621**	**0.587**	0.563	0.950	**0.504**	**0.638**	0.377
	P	0.296	**0.019**	0.063	**0.003**	0.745	0.688	0.449	0.832	0.125	0.630	**0.002**	**0.041**	0.435	0.497	**0.023**	**0.029**	0.813
	Random effect P	1.000	1.000	0.234	1.000	0.487	1.000	1.000	1.000	1.000	0.428	0.151	0.550	0.524	0.812	0.493	0.998	0.380

Gill raker number	Slope	0.360	0.114	-0.061	-0.100	0.065	**0.099**	-0.038	-1.267	0.004	0.025	**-0.453**	0.083	0.026	-0.045	-0.117	-0.073	0.015
	Std Error	0.233	0.109	0.118	0.058	0.079	**0.051**	0.152	2.016	0.102	0.064	**0.104**	0.080	0.072	0.131	0.078	0.073	0.057
	P	0.122	0.298	0.605	0.087	0.410	**0.050**	0.802	0.530	0.972	0.694	**0.000**	0.297	0.719	0.732	0.137	0.320	0.790
	Random effect P	1.000	0.995	**0.049**	0.999	0.479	1.000	1.000	0.000	1.000	0.428	0.151	0.550	0.485	0.812	0.097	0.998	0.380
Diet NMDS1	Slope			0.963	0.268	-0.477	0.061			0.445	-0.414		-0.314	0.543		0.296	**1.045**	-0.158
	Std Error			0.774	0.213	2.648	0.224			0.537	0.258		0.376	0.328		1.801	**0.422**	0.285
	P			0.214	0.210	0.857	0.784			0.407	0.109		0.404	0.098		0.869	**0.013**	0.578
	Random effect P			0.861	0.994	**0.000**	1.000			0.950	1.000		1.000	0.320		**0.000**	1.000	0.982
Diet NMDS2	Slope			-0.098	-0.319	-0.179	0.099			-0.835	-0.287		-0.680	-0.252		-0.974	-0.088	0.003
	Std Error			0.558	0.204	2.390	0.211			0.552	0.247		0.375	0.307		1.737	0.439	0.268
	P			0.861	0.117	0.940	0.638			0.131	0.246		0.069	0.413		0.575	0.841	0.990
Number of infected lakes		2	3	8	25	11	25	2	9	10	16	3	12	17	3	17	10	15

Table S3. General linear model results from host-population scale regressions of parasite prevalence on lake and population traits (e.g., trait means). We provide slope estimates and standard errors and P-values for the main effects listed. Positive and negative effects are shaded red and blue, respectively, for all significant (P < 0.05, also in bold font) and marginally significant (P < 0.1, not bold font) associations. Parasite taxa (columns) are ordered by average abundance across the entire metacommunity from most abundant (left) to least (right),

	Parasite taxon	Unionidae	Crepidostomum	Bunoderina	Thersitina	Dermocystidium	Diplostomum.sp1	Ergasilus	Neoechinorhyncus	Eustrongyloides	Schistocephalus.solidus	Diplostomum.spathaceum	Proteocephalus	Capillaria	Bunodera	Blackspot	Cystidicola	Anisakis	Neoechinocephalus	Glugea	Acanthocephalus
Elevation	Slope	0.002	-0.005	0.000	-0.008	-0.015	0.019	**-0.026**	0.004	-0.006	**0.036**	0.023	0.009	0.003	-0.007	-0.010	**0.042**	0.012	0.018	0.005	0.003
	Std Error	0.007	0.005	0.006	0.008	0.009	0.015	**0.011**	0.006	0.006	**0.017**	0.012	0.005	0.009	0.008	0.010	**0.018**	0.010	0.013	0.009	0.014
	P	0.735	0.394	0.946	0.290	0.099	0.235	**0.021**	0.475	0.346	**0.046**	0.069	0.119	0.749	0.425	0.308	**0.024**	0.222	0.172	0.570	0.841
Log lake area	Slope	**-0.404**	**-0.450**	0.021	-0.297	**-0.577**	-0.222	-0.096	-0.079	0.079	-0.159	-0.038	0.176	-0.047	0.011	0.210	0.103	0.066	-0.033	0.310	0.199
	Std Error	**0.174**	**0.142**	0.141	0.207	**0.260**	0.284	0.237	0.134	0.137	0.280	0.224	0.103	0.184	0.213	0.235	0.261	0.167	0.230	0.202	0.239
	P	**0.027**	**0.004**	0.880	0.162	**0.034**	0.441	0.688	0.561	0.570	0.575	0.865	0.098	0.799	0.961	0.378	0.697	0.694	0.887	0.136	0.412
Distance from ocean	Slope	0.028	0.050	0.002	0.032	0.076	0.061	**0.158**	-0.032	0.043	-0.013	**-0.107**	-0.009	0.028	0.016	0.036	0.026	0.027	0.024	-0.049	0.048
	Std Error	0.034	0.026	0.029	0.039	0.043	0.059	**0.054**	0.027	0.030	0.058	**0.050**	0.022	0.038	0.044	0.052	0.070	0.038	0.051	0.046	0.058
	P	0.415	0.061	0.948	0.419	0.089	0.309	**0.006**	0.253	0.157	0.819	**0.042**	0.690	0.462	0.712	0.499	0.711	0.488	0.643	0.288	0.418
Mean fish mass	Slope	**1.186**	0.347	-0.289	0.032	-0.528	3.112	0.103	-0.111	0.267	0.952	0.409	0.468	0.579	**1.615**	-0.400	**3.188**	-0.347	0.167	0.032	**1.481**
	Std Error	**0.492**	0.371	0.462	0.539	0.831	1.546	0.736	0.450	0.376	0.666	0.726	0.260	0.521	**0.487**	0.814	**1.157**	0.543	0.639	0.706	**0.732**
	P	**0.024**	0.359	0.537	0.954	0.531	0.055	0.890	0.808	0.484	0.165	0.578	0.084	0.276	**0.003**	0.627	**0.011**	0.529	0.797	0.964	**0.050**

Mean gape width	Slope	3.101	7.206	-3.023	**27.165**	2.558	24.265	10.589	-1.893	5.752	-3.976	**-17.130**	-5.659	4.562	2.720	4.113	-0.623	-6.135	-12.531	6.959	3.954
	Std Error	5.694	4.710	5.599	**9.532**	10.651	16.948	10.593	5.490	5.212	7.216	**7.983**	3.238	6.611	5.636	9.854	10.343	7.017	8.238	8.977	8.467
	P	0.591	0.139	0.594	**0.009**	0.812	0.165	0.327	0.733	0.280	0.587	**0.036**	0.093	0.497	0.634	0.680	0.952	0.390	0.141	0.445	0.645
Mean gill raker length	Slope	-3.542	-0.149	0.581	-4.226	-4.242	12.292	-2.343	0.823	-2.765	1.277	2.548	**-2.715**	0.080	1.896	3.892	-0.664	**-5.006**	**-6.502**	5.579	1.973
	Std Error	2.249	1.663	2.113	2.849	3.610	7.456	3.925	1.970	1.906	3.056	3.149	**1.278**	2.510	2.254	4.278	4.387	**2.438**	**3.082**	3.947	3.994
	P	0.128	0.929	0.785	0.150	0.251	0.112	0.556	0.680	0.159	0.680	0.426	**0.044**	0.975	0.408	0.372	0.881	**0.049**	**0.045**	0.170	0.626
Mean gill raker number	Slope	**0.762**	0.385	0.056	0.500	0.184	0.641	-0.788	0.415	0.252	-0.721	-0.495	0.210	0.268	**0.776**	-0.136	1.060	-0.372	-0.477	0.401	0.117
	Std Error	**0.295**	0.228	0.251	0.362	0.447	1.069	0.532	0.254	0.227	0.586	0.438	0.153	0.339	**0.317**	0.412	0.676	0.315	0.420	0.428	0.532
	P	**0.016**	0.103	0.826	0.179	0.683	0.554	0.151	0.115	0.277	0.230	0.269	0.182	0.437	**0.022**	0.744	0.129	0.249	0.266	0.357	0.827
Mean diet NMDS1	Slope	-0.094	2.023	**5.165**	-0.046	1.220	-6.352	-1.048	1.003	2.996	-2.982	**73.712**	-2.167	-0.977	-2.369	6.271	-1.048	2.248	-1.847	-0.025	-1.048
	Std Error	3.219	2.400	**2.137**	3.377	3.467	4.545	2.018	1.815	2.741	4.693	**15.349**	1.454	3.634	2.667	7.479	2.018	5.261	7.741	3.349	2.018
	P	0.977	0.410	**0.026**	0.989	0.729	0.179	0.610	0.587	0.289	0.533	**0.000**	0.153	0.791	0.386	0.413	0.610	0.674	0.814	0.994	0.610
Watershed random effect	P	0.498	0.453	0.807	0.344	0.851	0.000	0.672	0.206	0.977	0.007	0.164	0.194	0.743	0.028	0.291	0.092	0.462	0.456	0.466	0.400
