# Supplementary material for: Scale‐dependent effects of host patch traits on species composition in a stickleback parasite metacommunity
Source: Ecology. 2020 Oct 1;101(12):e03181. doi: 10.1002/ecy.3181 (PMC7757261; doi:10.1002/ecy.3181)
Supplement: Supplementary file 4 — Metadata S1 [file ECY-101-e03181-s004.pdf]

Bolnick, D.I., E.J. Resetarits, K. Ballare, Y.E. Stuart, W.E. Stutz. 2020. Scale-dependent effects of host patch traits on species composition in a stickleback parasite metacommunity. Ecology.

Data S1

The Data S1 supplement is a .zip folder containing the data files underlying results reported in the manuscript. These materials are also archived on Dryad at https://doi.org/10.5061/dryad.gmsbcc2j1 associated with another paper using these data for other purposes (Bolnick and Ballare 2020).

Authors of the material provided in DataS1.zip

Daniel I. Bolnick
Ecology and Evolutionary Biology & Institute of System Genomics, University of Connecticut, Storrs CT 06269, USA
Daniel.bolnick@uconn.edu

Emlyn J. Resetarits
Center for the Ecology of Infectious Disease, Odum School of Ecology, University of Georgia, Athens, GA, USA
Emlyn.resetarits@uga.edu

Kimberly Ballare
Ecology and Evolutionary Biology, University of California Santa Cruz, Santa Cruz, CA 95064, USA
Kim.ballare@gmail.com

Yoel E. Stuart
Department of Biology, Loyola University, Chicago IL, 60660, USA
ystuart@luc.edu

William E. Stutz
Office of Institutional Research, Western Michigan University, Kalamazoo, MI, USA
westutz@utexas.edu

File list (files found within DataS1.zip)

```
FishTraitData.csv
GeographicData.csv
IndividualFishHeterozygosity.csv
SNPgenotypes.csv
SNPlocusinformation.csv
SNPmetadata.csv
```

Description

The following data files are provided in this supplement, in comma-separated value .csv format:

Filename: `FishTraitData.csv`
Description: The meta-data table for trait data on individual fish. 4376 rows, 172 columns. Each row represents an individual fish, columns are data concerning that individual. Missing values (not all fish were scored for all variables) contain NA. Columns include:
A. site_name: name of the water body from which the fish was collected
B. habitat_type: Estuary, Stream, Lake
C. watershed
D-E. GPS location of collection in UTM, North and East
F-G. Collection date and year
H-I. Metadata identifiers for internal bookkeeping
J. Lake elevation
K. Lake maximum depth
L. Lake surface area
M. Lake permieter
O. Lake distance from ocean
P. BC_ID Waterbody identifier
Q. Individual conducting measurements
R. Measurement date
S. Notes on collection location
T. Trap: a unique identifier on a given collection day, to distinguish fish collected in different traps.
U. sex (F/M)
V. mass (g)
W. Standard length std_length (mm)
X-AA. Morphological traits measured on a subset of individuals, including body depth (mm), body width (mm), gape width (mm), gill raker length (normalized)
AB-AF. Log transforrmed morphological traits
AG. Gill raker length raw values in ocular micrometer units
AH-AI. Left and right armor plate counts

AJ. Gill raker count.
AK-AO. Residual trait values from regressions on standard length (after trait ln transformation)
AQ-AU. Morphological PCA scores
AV-BG. Geometric morphometric scale, centroid size, and PC axis scores from photographs.
BI-CX. Parasite counts
CY. Parasite richness
CZ Parasite shannon-weaver diversity index
DA. Relativized infection intensity
DB-DC. Parasite nestedness scorre
DD-DH. Parasite NMDH score
DL- FA. Presence/absence (1/0) of potential diet items in stomach contents.
FB. Number of prey types
FC-FG. Diet PCA scores
FH-FI. Nestedness of diet
FJ. Proportion benthic prey in diet
FJ-FO diet NMDS scores

Filename: `GeographicData.csv`
Description: Meta-data on lakes and collection sites. 56 rows (sites) by 67 columns in a .csv file. Missing values contain NA. Columns include:
A. counter
B. site_name
C. habitat_type (Estuary, Stream, Lake)
D.E. Latitude/ Longitude coordinates in UTM north and east (all zone 10)
F. Elevation. meters
G. Maximum depth (NA for streams or ocean/estuary). Meters
H. Surface area (NA for streams or ocean/estuary). hectares
I. Perimeter (NA for streams or ocean/estuary). kilometers
J. Other species recorded in BCFishBase
K. Watershed
L. Waterbody ID Code for BC MoE
M. distance_from_ocean_km
N. Date sampled in 2009
O. chlorophyll_a_ ug/L
P. Water: marine/fresh
Q. Secchi depth
R. Thermocline depth (m)
S. Mean temperature (C)
T. Mean Oxygen concentration (percent saturation)
U. Mean salinity (microSeimens)

V. Date sampled
W. Depth at water chemistry sampling site
X-AN. Zooplankton biomass per L (mg/L) from plankton tows
AO-AQ: plankton NMDS Scores 1-3
AR-BO: mean and standard deviation length measures on plankton species.

Filename: `IndividualFishHeterozygosity.csv`
Description: Mean genomic heterozygosity per individual for 12 fish per site. A .csv file containing 336 row, 5 columns:
A. Counter
B. FishID to match to FishTraitData.csv
C. Site name
D. Watershed
E. Habitat type (L = lake, S = stream, M = marine)
F. Mean genomic heterozygosity for the scored SNPs. By using SNPs, this overestimates genome-wide heterozygosity but works as a relative measure across lakes or among individuals. To obtain this number we calculate heterozygosity for every SNP scored in the data set for every individual. Then we average across scored sites, for each individual.

Filename: `SNPgenotypes.csv`
Description: Individual fish genotypes for all scored SNPs. Each fish is a row, each SNP is a column (1176 rows, 244,315 columns). This is a .csv file. Row information is stored in SNPmetadata.csv. Column information is stored in SNPlocusinformation.csv. Individual genotypes are scored as 0 (reference genome allele), 1 (heterozygote), or 2 (homozygote with an alternate allele). The first column is an identifier, followed by columns for 244,314 SNPs.

Filename: `SNPlocusinformation.csv`
Description: Information on the SNPs that form the columns in SNPgenotypes.csv. 244,314 rows and 11 columns of metadata. Columns include:
V1: counter
SNPnames: names to match column names in SNPgenotypes.csv
group: linkage group number; FALSE if mitochondrial or scaffold SNP
MT: True if mitochondrial, False otherwise
scaffold: True if unassembled scaffold (not assigned to a chromosome, False otherwise)
Chromosomal_pos: SNP position along the length of the linkage group in question
Original_order: for bookkeeping
genomic_order: for bookkeeping
Chr_end: for bookkeeping
cumulative_xpos: cumulative genomic position, starting with mitochondrial sequence then LG1, LG2..... scaffolds.

Filename: `SNPmetadata.csv`
Description: Data on individual fish (rows) corresponding to SNPgenotypes.csv, in the same order. Columns include:
A. Counter
B. Watershed
C. Habitat (S/L/M)
D. Site (lake or stream name)
E. FishID. This matches data in the FishTraitData.csv file, while the row order in this file matches SNPgenotypes.csv row order, allowing you to connect SNP genotypes to fish traits.

Associated data sets stored elsewhere:
The original raw sequence reads are archived by Stuart et al. 2017 Nature Ecology & Evolution.

Whom to contact with questions:
Dr. Daniel Bolnick
Department of Ecology and Evolutionary Biology
University of Connecticut
Storrs CT
daniel.bolnick@uconn.edu
512-809-6217

Literature Cited

Bolnick, D.I. and Ballare, K.M. (2020b), Resource diversity promotes among-individual diet variation, but not genomic diversity, in lake stickleback. Ecology Letters 23:495–505.

Stuart, Y. E., T. Veen, J. N. Weber, D. Hanson, B. K. Lohman, C. J. Thompson, T. Tasneem, A. Doggett, R. Izen, N. Ahmed, R. D. H. Barrett, A. P. Hendry, C. L. Peichel, and D. I. Bolnick. 2017. Contrasting effects of environment and genetics generate a continuum of parallel evolution. Nature Ecology and Evolution 1:0158.
