# Supplementary material for: Scale‐dependent effects of host patch traits on species composition in a stickleback parasite metacommunity
Source: Ecology. 2020 Oct 1;101(12):e03181. doi: 10.1002/ecy.3181 (PMC7757261; doi:10.1002/ecy.3181)
Supplement: Supplementary file 5 — Metadata S2 [file ECY-101-e03181-s005.pdf]

Bolnick, D.I., E.J. Resetarits, K. Ballare, Y.E. Stuart, and W.E. Stutz. 2020. Scale-dependent effects of host patch traits on species composition in a stickleback parasite metacommunity. Ecology

---

Data S2

Results of testcov() comparisons of parasite-parasite cooccurrence matrices between pairs of lakes (co-occurrence at the individual host scale), and in comparison to the among-lake covariance matrix (second column on the right), and the covariance matrix among all fish (omitting population structure, right-most column). Cells present p-values testing the null hypothesis that a given comparison has the same underlying covariance matrix.

Authors of the material provided in DataS2.zip

Daniel I. Bolnick (Author to contact with questions)
Ecology and Evolutionary Biology & Institute of System Genomics, University of Connecticut, Storrs CT 06269, USA
Daniel.bolnick@uconn.edu

Emlyn J. Resetarits
Center for the Ecology of Infectious Disease, Odum School of Ecology, University of Georgia, Athens, GA, USA
Emlyn.resetarits@uga.edu

Kimberly Ballare
Ecology and Evolutionary Biology, University of California Santa Cruz, Santa Cruz, CA 95064, USA
Kim.ballare@gmail.com

Yoel E. Stuart
Department of Biology, Loyola University, Chicago IL, 60660, USA
ystuart@luc.edu

William E. Stutz
Office of Institutional Research, Western Michigan University, Kalamazoo, MI, USA
westutz@utexas.edu

File list (file found within DataS2.zip)

testcov_comparisons.csv

Description

testcov_comparisons.csv – Results of testcov() comparisons of parasite-parasite cooccurrence matrices between pairs of lakes (co-occurrence at the individual host scale), and in comparison to the among-lake covariance matrix (second column on the right), and the covariance matrix among all fish (omitting population structure, right-most column). Cells present p-values testing the null hypothesis that a given comparison has the same underlying covariance matrix.
